# New carnivorous sponges (Porifera: Cladorhizidae) from Western Australia, collected by a Remotely Operated Vehicle (ROV)

**DOI:** 10.1038/s41598-024-72917-8

**Published:** 2024-09-27

**Authors:** Merrick Ekins, Nerida G. Wilson

**Affiliations:** 1 Queensland Museum, South Brisbane, PO Box 3300, Brisbane, Queensland 4101 Australia; 2 School of Biological Sciences, University of Queensland, St Lucia, Queensland 4072 Australia; 3 Griffith Institute for Drug Discovery, Griffith University, Brisbane, Queensland 4111 Australia; 4 Research & Collections, Western Australian Museum, 49 Kew Street, Welshpool, Western Australia 6106 Australia; 5 School of Biological Sciences, University of Western Australia, 35 Stirling Hwy, Crawley, Western Australia 6009 Australia

**Keywords:** *Abyssocladia*, *Axoniderma*, *Cladorhiza*, *Nullarbora*, Ningaloo, Cape range canyon, Bremer canyon system, Indian ocean, Schmidt ocean institute, Zoology, Taxonomy

## Abstract

The last two decades have reinvigorated systematic research on predatory sponges, mainly fuelled by advances in technology that have facilitated collection in deep-water habitats. This research presents six new species of carnivorous sponges from the family Cladorhizidae Dendy, 1922 from the western continental margin of Australia. The new species are *Abyssocladia johnhooperi* nov. sp., *Abyssocladia aurora* nov. sp., *Abyssocladia janusi* nov. sp., *Axoniderma challengeri* nov. sp., *Cladorhiza vanessaekins* nov. sp*.* and *Nullarbora ningalooa* nov. sp.. This material was collected by ROV during expeditions FK200308 to the Ningaloo Canyons expedition off the mid-west coast near Ningaloo, and FK200126 to the Southwest Australian canyons expedition, in Western Australia. These and other expeditions by the Schmidt Ocean Institute in 2020–21 formed a campaign around Australia’s deep sea and mesophotic environments, which has vastly increased our understanding of biodiversity in these habitats.

## Introduction

Prior to the discovery of carnivory in sponges^1^, the collection of cladorhizid sponges was through trawls along the oceans floor (including the Indian Ocean) and retrieval by piano wire 150 years ago during the Challenger Expedition (1872-1876), later beautifully described by Ridley & Dendy^2,3^. The last two decades, however, has seen an explosion in the discovery and description of new species. This has been largely driven by the development of ROV’s capable of reaching bathyal and abyssal depths where these species commonly live.

The family Cladorhizidae, which contains the carnivorous sponges, underwent some significant recent re-arrangement. As an example, all of the species previously encompassed within the genus *Cladorhiza* were compared^4^ and then split^5^ into five genera, some of which were new and some resurrected genera. These now include *Cladorhiza* Sars 1872 (arbuscular morphology), *Axoniderma* Ridley & Dendy, 1886 (classic parasol/umbrella morphology), *Abyssosdiskos* Ekins, Erpenbeck, Goudie & Hooper, 2020 (upwards facing disc), *Bathytentacular* Ekins, Erpenbeck, Goudie & Hooper, 2020 (teardrop with tentacular processes) and *Nullarbora* Ekins, Erpenbeck, Goudie & Hooper, 2020 (pinnate morphology).

The RV *Falkor* carried out a series of cruises around Australia in 2020/2021, which produced significant new information on the diversity of carnivorous sponges. One of those expeditions, FK200802—Seamounts, Canyons & Reefs of the Coral Sea Cruise, discovered three new species *Abyssocladia falkor* Ekins & Hooper, 2023, *A. jeanvaceleti* Ekins & Hooper, 2023 and *Axoniderma wanda* Ekins & Hooper, 2023. In addition, two other Australian species of carnivorous sponge *Chondrocladia* (*Chondrocladia*) *zygainadentonis* Ekins et al., 2020a and *Asbestopluma* (*Abestopluma*) *maxisigma* Ekins et al., 2020a, previously known from the east coast of Australia were redescribed. The total known fauna of Cladorhizidae in Australian waters consists of 35 species.

Previous collections around Australia have not included Western Australia. However, there have been several reports of carnivorous sponges collected from the Indian Ocean starting with the Challenger expedition. From the Indian Ocean leg of the cruise, Ridley & Dendy^2,3^ described *Cladorhiza moruliformis* Ridley and Dendy, 1886, *Cl. tridentata* Ridley and Dendy, 1886, *Abyssocladia symmetrica* (Ridley and Dendy, 1886) and *Chondrocladia* (*Melliderma*) *stipitata* Ridley and Dendy, 1886. From the Indian Ocean “Galathea” expeditions, Lévi^6^ described *Cl. ephyrula* Lévi, 1964, *Cl. nematophora* Lévi, 1964, *Ch.* (*Ch.*) *multichela* Lévi, 1964, *Ch*. (*Ch*.) *dichotoma* Lévi, 1964, *Ch*. (*Ch*.) *gracilis* Lévi, 1964, and a description of a new specimen of *Ch*. (*Ch*.) *clavata* Ridley and Dendy, 1886.

From the more ubiquitous Southern Ocean area, which may overlap with some Indian Ocean fauna, Topsent^7^ described *Cladorhiza thomsoni* Topsent, 1909 from an island on the South Atlantic Ocean off Cape Town, South Africa. Similarly, Koltun^8^, described *Cl. mani* Koltun, 1964, from the Southern Ocean south of South Africa, and described *Cl. tridentata.* From the Eastern Antarctic (Indian Ocean), Koltun^8^ described *Asbestopluma* (*Asbestopluma*) *obae* Koltun, 1964 and included descriptions of *Chondrocladia* (*Ch*.) *antarctica* Hentschel, 1914, *Cl*. *moruliformis*, *Lycopodina callithrix* (Hentschel, 1914), *L. calyx* (Hentschel, 1914) and *As*. (*As*.) *belgicae*. From the Kerguelen Islands in the Indian Ocean, two species were described^9^*Ch*. (*Ch*.) *fatimae* Boury-Esnault & van Beveren, 1982 and *Ch*. (*Ch*.) *nani* Boury-Esnault & van Beveren, 1982, and new material of *Ch*. (*Ch*.) *clavata* was described.

In more extensive recent work, Hestetun et al.^10^ described nine new cladorhizid species in the Southwest Indian Ocean Ridge seamounts collected by ROV on RRS *James Cook* cruise JC066 in 2011. The new species described were *Abyssocladia boletiphora* Hestetun, Rapp & Xavier, 2017, *A. corniculiphora* Hestetun, Rapp & Xavier, 2017, *A. hemiradiata* Hestetun, Rapp & Xavier, 2017, *Asbestopluma* (*Asbestopluma*) *unguiferata* Hestetun, Rapp & Xavier, 2017, *As.* (*As*.) *jamescooki* Hestetun, Rapp & Xavier, 2017, *As*. (*As*.) *laminachela* Hestetun, Rapp & Xavier, 2017, *As*. (*As*.) *pseudoisochela* Hestetun, Rapp & Xavier, 2017, *As*. (*As*.) *ramuscula* Hestetun, Rapp & Xavier, 2017 and *Chondrocladia* (*Meliiderma*) *rogersi* Hestetun, Rapp & Xavier, 2017. Hestetun et al.^10^ included a map of the Indian Ocean showing the distribution of species previously recorded from the Indian Ocean, and Southern Atlantic Ocean and species from East Antarctica. Historical Indian Ocean species collected by the Challenger Expedition and described by Ridley and Dendy^2,3^ i.e. *A. symmetrica*, *Ch*. (*M*.) *stipitata*, *Cladorhiza moruliformis* and *C. tridentata* were redescribed from the type material by Hestetun et al.^10^. Most recently, Rengaiyan & Ingole^11^, described three new carnivorous sponges, *As.* (*As.*) *indiyansis* Rengaiyan & Ingole, 2022, *As.* (*As*.) *bharatiyae* Rengaiyan & Ingole, 2022 and *Ch. sagari* Rengaiyan & Ingole, 2022, from the Central Indian Ridge collected by trawl.

**Fig. 1:**
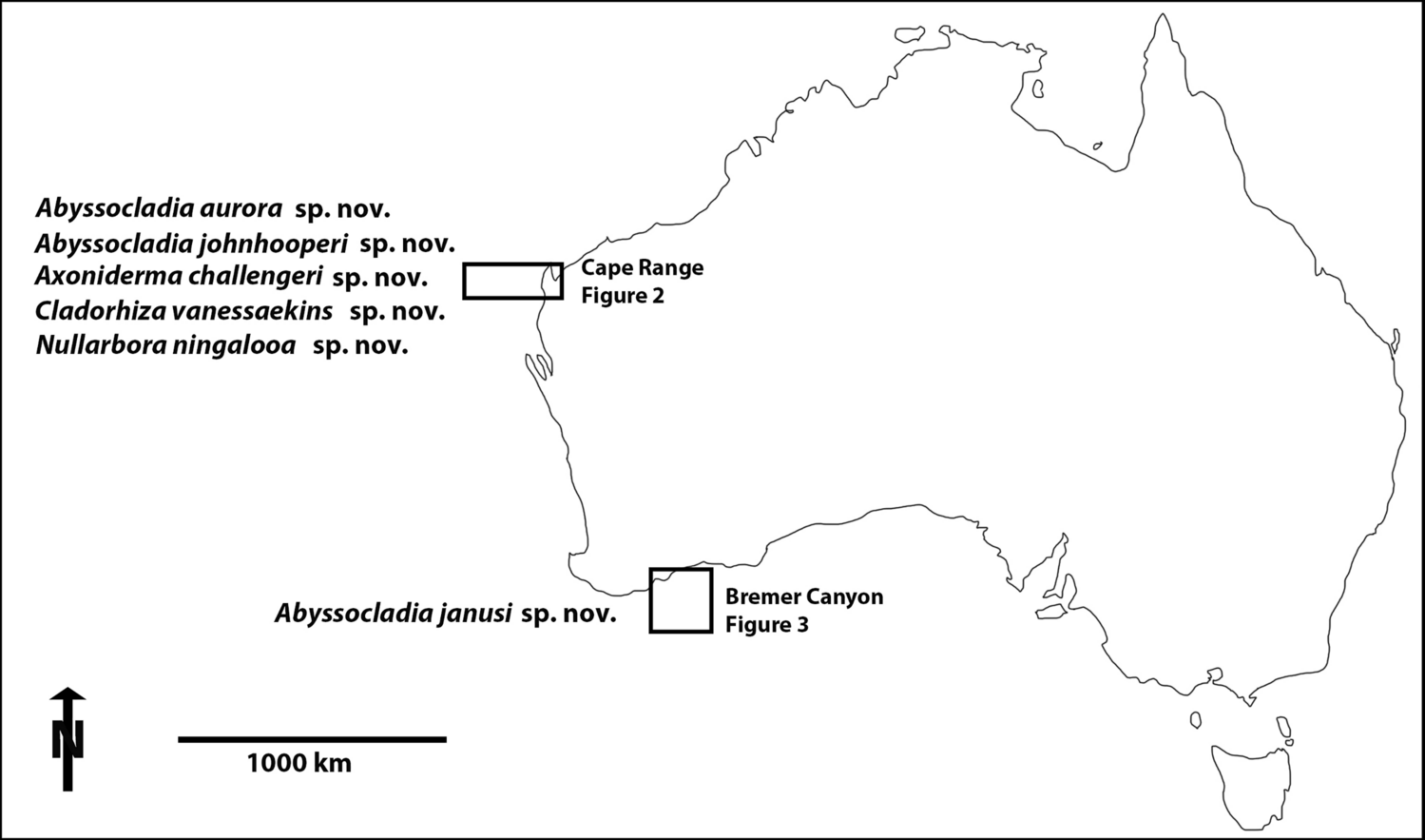
Collection locations of the new species of carnivorous sponges created in Adobe Photoshop.

Here we report on an additional six new species of carnivorous sponges discovered from two deep-sea localities in Western Australia, separated by almost a thousand kilometres, and several thousand kilometres from any other Indian Ocean carnivorous sponge records. The scientific expeditions to these two locations are the Ningaloo Canyons expedition FK200308^12^ and the Southwest Australian canyons expedition FK200126^13^ (Fig. 1)

## Materials and methods

**Fig. 2:**
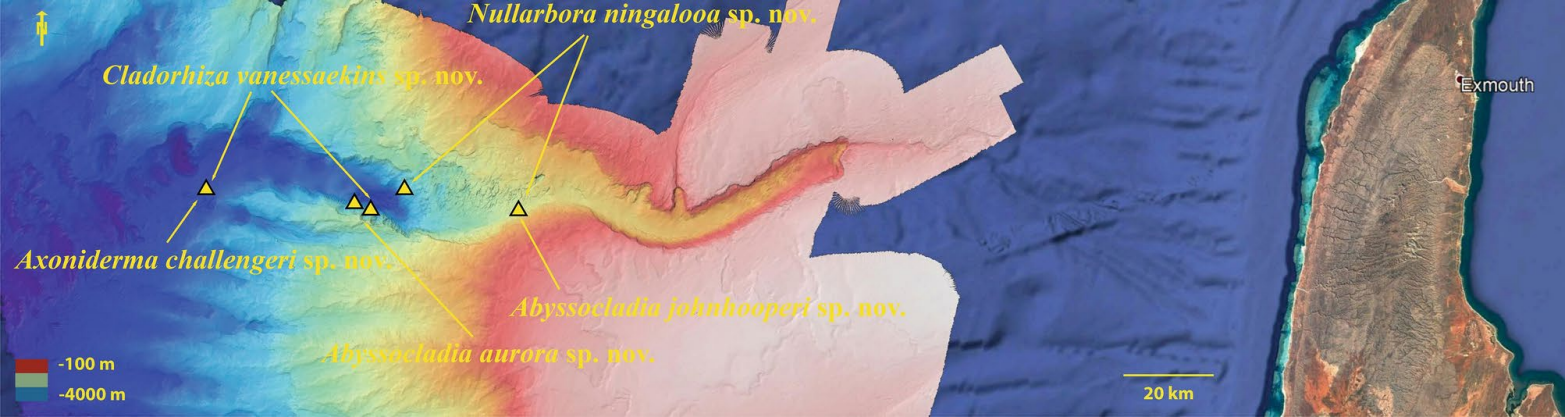
False colour bathymetry map of FK200308 showing the dive locations where species were described from Cape Range Canyon, created in Kongsberg EM 302 and 710 multibeam sonar system, then overlaid on Google Earth and assembled in Adobe Photoshop.

**Fig. 3:**
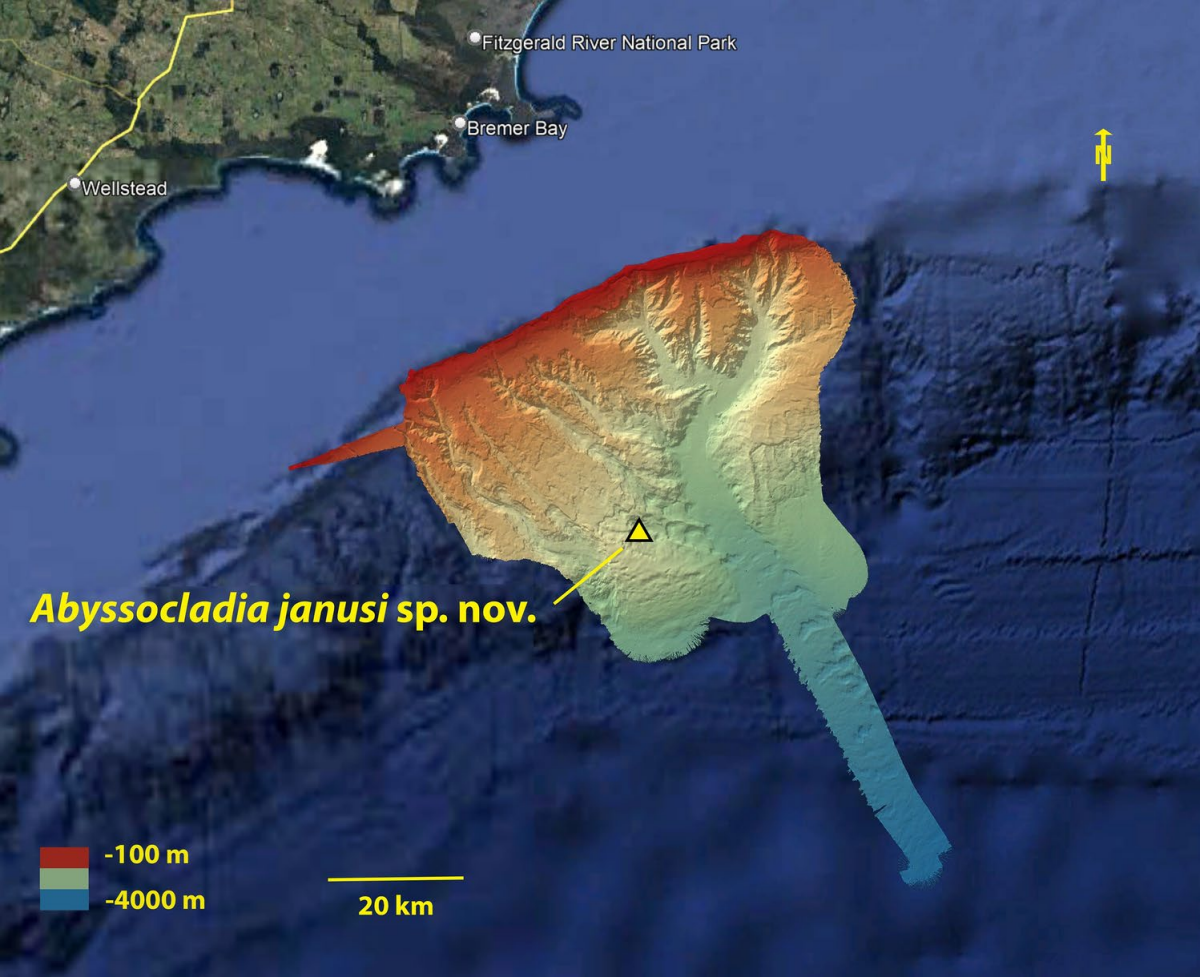
False colour bathymetry map of FK200126 showing the dive location from the Bremer Canyon system, created in Kongsberg EM 302 and 710 multibeam sonar system, then overlaid on Google Earth and assembled in Adobe Photoshop.

The material described here was collected by the ROV *SuBastian* aboard the Schmidt Ocean Institute’s research vessel RV *Falkor* during the FK200308 Ningaloo expeditions and FK200126 Southwest Australian canyons expedition (Figs. 1, 2, 3). The bathymetry maps were generated using the RV *Falkor*’s multibeam sonar systems (Kongsberg EM 302 and 710) then overlaid on Google Earth. All of the figures were created and/or assembled in Adobe Photoshop. The specimens were collected with either manipulator jaws or suction, stored in seawater, photographed and then immediately transferred to 70 % ethanol, with tissue subsamples taken into 96% ethanol.

### Morphological analysis

Scanning Electron Microscope (SEM) spicule preparations were made by dissolving soft tissue in 12.5% sodium hypochlorite, neutralized in distilled water, rinsed again in distilled water, rinsed twice in 70% ethanol and then finally rinsed twice in 98% ethanol and air dried. SEM preparations were sputter coated in gold to improve resolution. The SEM micrographs and measurements were made using a Hitachi TM-1000 SEM and plates assembled in Adobe Photoshop. The spicule size ranges are from those spicules that were the largest and smallest of all spicules from all specimens. Statistics were compiled in Mircosoft Excel. Specimens in the laboratory were photographed using a Canon EOS 7D. Specimens were examined under an Olympus SZ60 dissection microscope and photographed with a Tucsen 3.0 camera.

### Molecular analysis

DNA was extracted from tissue samples using the DNeasy Blood & tissue kit (Qiagen) following the manufacturer’s protocol. The C-region of the 28S rRNA gene, recommended for sponge barcoding^14^, was amplified using primers 28S-C2-fwd and 28S-D2-rev^15^. All available Cladorhizidae 28S sequences on GB were downloaded (19 Dec 2022) and aligned with newly-generated data, along with outgroups from the genus *Mycale* Gray, 1867. The alignment was generated using the MAAFT^16,17^ v7.490 plugin to Geneious using the E-INS-I algorithm with flanks manually trimmed. One sequence was removed because it was very short (AY348890) and five others^18^ could not be aligned with the rest of the data set. The alignment was analysed using Maximum Likelihood in IQ-tree^19^ web server using 1000 ultrafast bootstrap replicates^20^ to assess node support. The tree file was formatted in FigTree v1.4.4 and exported as a pdf.

## Results

### Genus *Abyssocladia* Lévi, 1964

*Abyssocladia* Lévi, 1964: 78

**Diagnosis**. Cladorhizidae most often pedunculate, carrying a disciform or flabelliform body with a radial architecture, in other cases pinnate or branching. Microscleres are a combination of abyssochelae, cleistochelae, arcuate chelae and/or sigmancistras, but not placochelae^21^.

**Type species**. *Abyssocladia bruuni* Lévi, 1964 (by monotypy).

**Remarks**. Currently there are 40 accepted species assigned to *Abyssocladia*^22^, with only five previously recorded from the Australian EEZ i.e. *A. annae* Ekins, Erpenbeck & Hooper, 2020a, *A. desmophora* (Hooper & Lévi, 1989), *A. escheri* Ekins, Erpenbeck & Hooper, 2020a, *A. falkor*, *A. gliscofila* Ekins, Erpenbeck & Hooper, 2020a, *A. jeanvaceleti* and *A. oxyasters* Ekins, Erpenbeck, Goudie & Hooper, 2020b. Two new of *Abyssocladia* were recently recorded from New Caledonia^23^. They are *A. kanaconi* Vacelet, 2020 and *A. microstrongylata* Vacelet, 2020.

### ***Abyssocladia johnhooperi* sp. nov.**

**Fig. 4:**
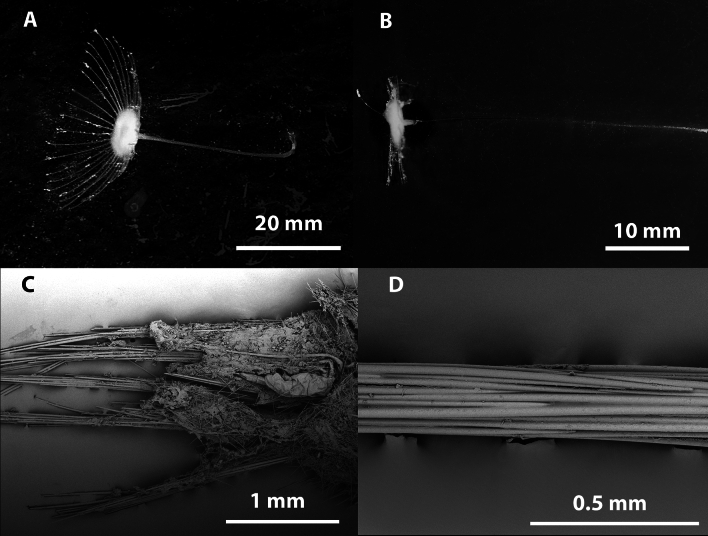
*Abyssocladia johnhooperi* sp. nov. (**A**). Holotype WAMZ100664 in situ before collection. (**B**). Holotype after collection. (**C**). SEM of the body face and filaments emerging, with ensnared isopod prey. (**D**). SEM of stem showing the axial bundles of styles.

**Fig. 5:**
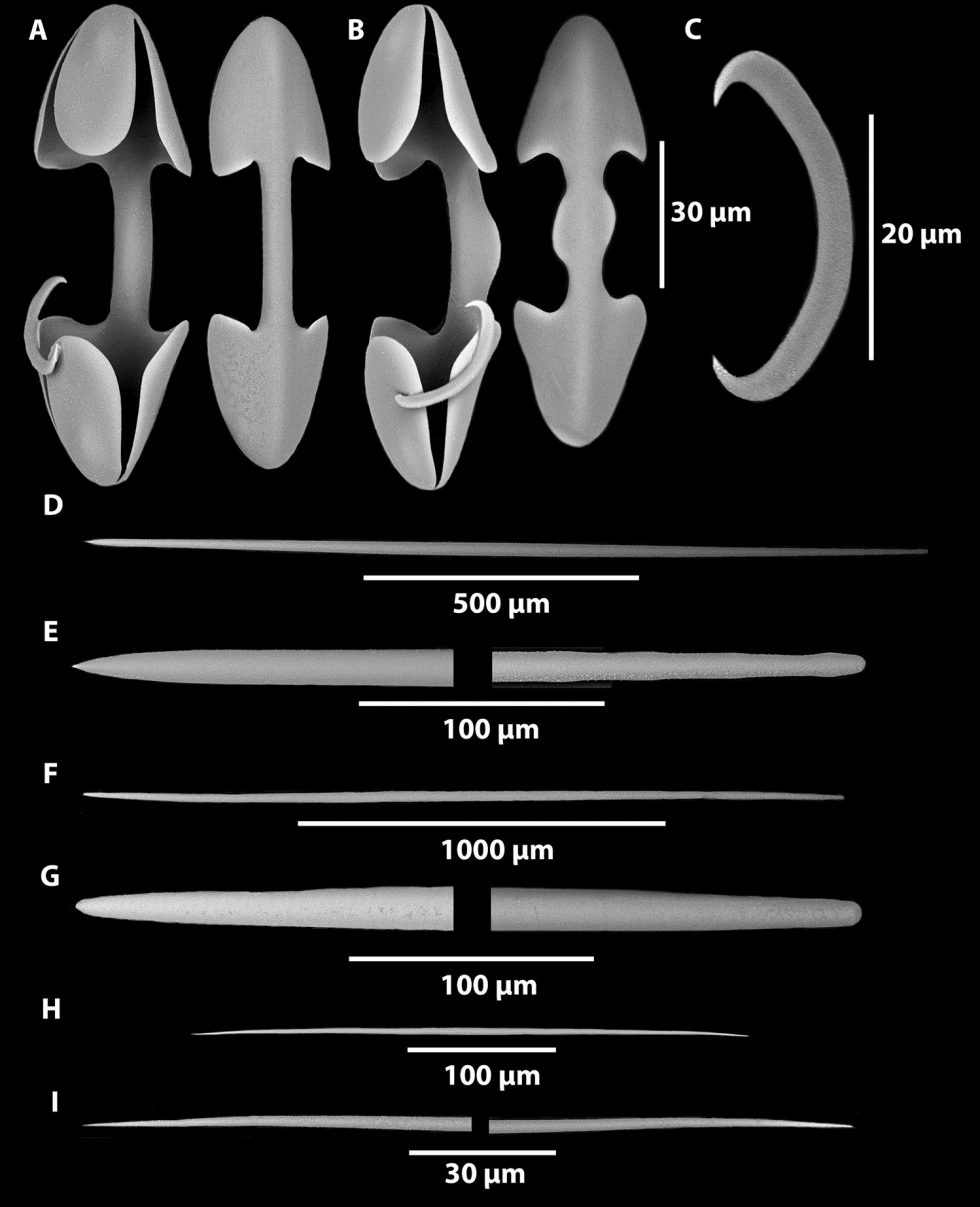
SEM of *Abyssocladia johnhooperi* sp. nov. (**A**). Isochelae. (**B**). Isochelae with central saddle. (**C**). Sigmancistras. (**D**). Mycalostyles from the filaments with subtle subtylostyle swelling. (**E**). Magnified ends of the mycalostyle illustrated in (**D**) with subtle subtylostyle swelling. (**F**). Mycalostyles from the stem. (**G**). Magnified ends of the mycalostyle illustrated in (**F**). (**H**). Thin oxeas in the filaments. (**I**). Magnified ends of the oxeas illustrated in (**H**).

**Fig. 6:**
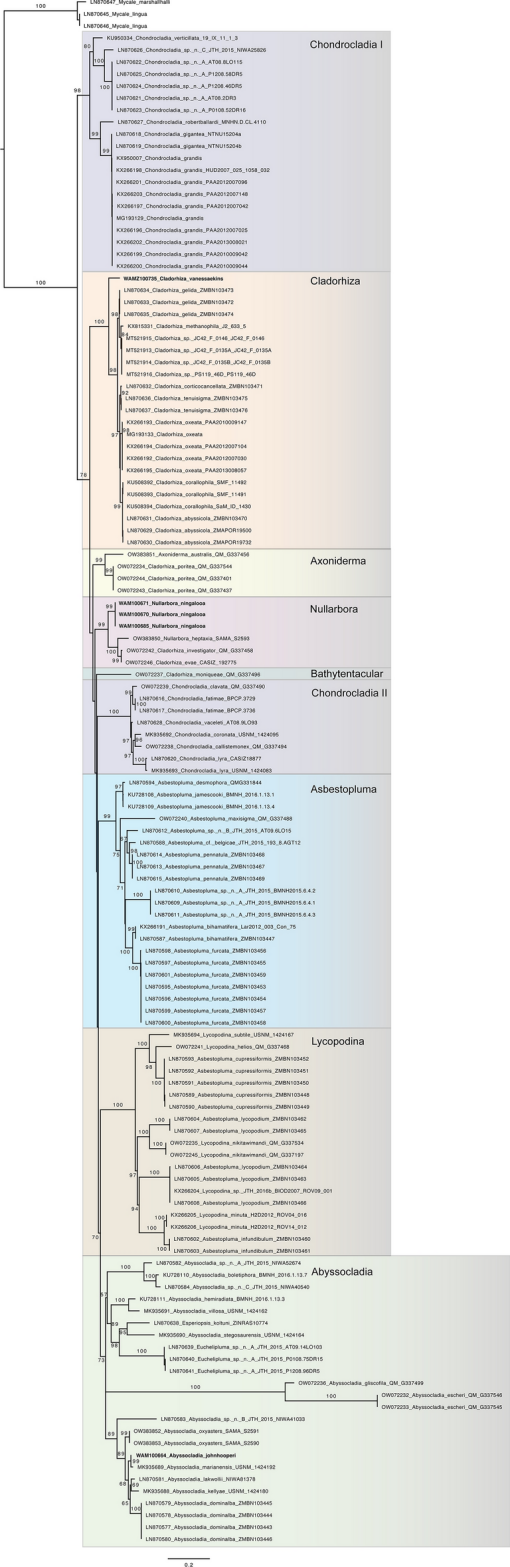
Maximum-likelihood tree of new and available Cladorhizidae 28S-C sequences.

Figs. 4, 5, 6, Table 1.

**Table 1:** Comparative morphological and distributional data for all staked disc-shaped species of *Abyssocladia*.

Species	Reference	Morphology	Total height x width (mm)	Skeleton	Spicules of main axis (LxW um)	Spicules of lateral filaments or body (LxW um)	Spicules of basal attachment (LxW um)	Chelae (L um)	Sigmancistras (L um)	Sigmas (L um) and other microscleres	Locality, depth range
*Abyssocladia johnhooperi* sp. nov	This work	Pedunculate, erect, long thin stem with convex disc shaped body on apex encircled by radiating curved filaments	47 × 35	Dense bundles longitudinally arranged styles. The main body and filaments consists of the radiating bundles of subtylostyles and oxeas that form the filaments	Mycalostyles 1030–2380 × 16–32	Subtylostyles 1080–1670 × 15–34 Oxeas 151–530 × 2–6	Base missing	Palmate isochelae 60–121 × 3–9	21–33 × 1–3	Absent	Ningaloo, Western Australia, abyssal
*Abyssocladia aurora* sp. nov	This work	Peduncle, erect long thin stem bearing a vertically orientated ovoid body with filaments radiating in a single plane	36 × 10	Dense bundles longitudinally arranged styles. The main body and filaments consists of the radiating bundles of mycalostyles and styles that form the filaments	Mycalostyles 936–2450 × 15–38	Mycalostyles 936–2450 × 15–38 styles 98–218 × 2–3	Strongyles 471–1030 × 20–36	Abyssochelae 47–142	21–31 × 1–3	Sigmas 30–55 × 1–2	Ningaloo, Western Australia, abyssal
*Abyssocladia janusi* sp. nov	This work	Pedunculate, erect, long thin stem with multiple concave faced body on apex, Each face encircled by radiating curved filaments	55 × 20	Dense bundles longitudinally arranged styles. The main body and filaments consists of the radiating bundles of mycalostyles and oxeas that form the filaments	Mycalostyles 1730–2390 × 21–50	Mycalostyles 1730–2390 × 21–50 Oxeas 422–623 × 3–6	Base missing	Palmate isochelae 47–72 × 2–4	28–36 × 2–4	Absent	Bremer Canyon system, Western Australia, abyssal
*Abyssocladia falkor* Ekins, & Hooper, 2023	Ekins, & Hooper, 2023:440-443, Figs 4-5	Pedunculate short erect stem and a vertically orientated disc shaped body with filaments radiating out in a single plane from the disc margin	50 × 10	Dense longitudinal bundles of mycalostyles and styles in the axis of peduncle, body with radial skeleton, bundles of mycalostyles extending into filaments, roots strongyle bundles	Mycalostyles 715–1020 × 10.0–30.7, oxetote styles 282–621 × 6.4–14.7, sinuous styles 222–540 × 4.9–13.5	Subtylostyles 192–830 × 3.2–12.3	Strongyles 220–490 × 5.5–14.7	Abyssochelae 27.5–52.8 × 1.9–8.6	6.9–10.9 × 0.5–1.8	Sigmas I 50.6–67.2 × 0.8–1.5, sigmas II 16.8–33.1 × 1.1–3.1, spherical microstrongyles 8.4–26.0 × 5.8–12.9, tylostyle microtrongyles 9.0–73.3 × 2.8–8.5	Great Barrier Reef, Queensland, Australia, bathyal
*Abyssocladia jeanvaceleti* Ekins, & Hooper, 2023	Ekins, & Hooper, 2023:443-452, Figs 6-7	Pedunculate short erect stem and a vertically orientated disc shaped body with filaments radiating out in a single plane from the disc margin	40 × 30	Dense longitudinal bundles of mycalostyles in the axis of peduncle, body with radial skeleton, bundles of mycalostyles extending into filaments, roots strongyle bundles	Mycalostyles I 620–1620 × 12.6–29.8 mycalostyles II 387–962 × 19.1–35.1	Mycalostyles I 738–1710 × 11.9–26.8 mycalotyles II 364–898 × 5.0–12.9	Strongyles 315–678 × 7.5–26.3	Palmate Isochelae 24.5–61.4 × 3.5–6.0	8.1–26.9 × 0.6–2.0	Absent	Great Barrier Reef, Queensland, Australia, bathyal
*Abyssocladia annae* Ekins, Erpenbeck & Hooper 2020	Ekins, Erpenbeck & Hooper 2020:23–25, Fig 4	Pedunculate erect stem supporting a slightly cupped-shaped obovate (leaf- like, flabellate, fan-shaped) apical body, lacking lateral filaments	3 × 2	Peduncle axis longitudinally arranged subtylostyles, body with radiating subtylostyles projecting only in one quadrant	Subtylostyles 288–1000 × 3.1–15.2	Undifferentiated	Base missing	Abyssochelae 48.2–72.2 × 5.1*–*14.9	11.6—(15.6)-18.5	Absent	Off the continental shelf of central New South Wales, Australia, abyssal
*Abyssocladia carcharias* Kelly & Vacelet, 2011	Kelly & Vacelet, 2011:58–60, Figs 2-3	Pedunculate, long thin peduncle, flattened circular body, with short blunt radiating filaments	7 × 3.4	Dense longitudinal bundles of mycalostyles in the axis of peduncle, body with radial skeleton, bundles of mycalostyles extending into filaments	Mycalostyles 510–1070 x 8–19 microstyles 140–240 x 2.5–5	Undifferentiated	Base missing	Unguiferate abyssochelae 1, 116–197 abyssochelae 2, 60–86 abyssochelae 3, 35–48	Sigmancistras 1, 15– 16.2 × 1.9– 2 sigmancistras 2, 8– 128–12	Absent	Kermadec Seamounts, New Zealand, bathyal
*Abyssocladia dominalba* Vacelet, 2006	Vacelet, 2006: 575–577,Fig. 14	Long thin pedunculate with an ovoid or subspherical body, body with dense spicular bundles laterally radiating	28–31	Peduncle axis with longitudinally arranged long fusiform styles, supported by radiating bundles of fusiform styles and smaller styles with tips directed outwards	Styles 620– 2500 × 7–35	Undifferentiated	Base missing	Arcuate isochela 80–170 abyssochelae—cleistochelae 40–45 anisochelae 9.5–11	1, 30–40 2, 9.5–12.5	Absent	North- Fijian back-arc Basin, bathyal
*Abyssocladia fryerae* Hestetun, Rapp & Pomponi, 2019	Hestetun, Rapp & Pomponi, 2019:4, Fig. 2	Pedunculate, short stem, disc-shaped droplet-like body, radiating long filaments in a single plane from disc margin	25 × 12	Stem with tightly packed subtylostyles entering center of disc-shaped body with radial skeleton, filaments and disc also composed of subtylostyles	Subtylostyles 582–1130 x 3.8–23.5	Undifferentiated	Undifferentiated	Arcuate isochelae 77.9–110.3	17.9–22.9	Absent	Marianas, NW Pacific, abyssal
*Abyssocladia huitzilopochtl i* Vacelet, 2006	Vacelet, 2006:569–573, Figs 9-11	Erect pedunculated, enlarged base, long thin peduncle, flattened semicircular disc-like body, with numerous free radiating spicule fascicles protruding	41 × 0.3-0.6	Axis of peduncle tightly packed with longitudinal bundles of long substrongyles, body with radiating bundles and irregularly dispersed large substrongyles, filaments with smaller bundles of substrongyles	Styles—substrongyles 1 (peduncle, filaments), 1050–2500 x 15–30, substrongyles 2 (body, filaments), 260–660 × 5–10	Undifferentiated	Substrongyles 3 (base), 560–750 x 21–30	Abyssochelae (body, upper stem) 60–80 arcuate isochelae 1 (body, filaments), 67–90 arcuate isochelae 2 (base of stem), 40–55	Sigmancistras1, 20–24 2, 11–12 orthancistras (body, filaments) 150–195	Microxeas (possibly foreign) 30–95 × 0.3–1	Middle America Trench, off Mexico, bathyal
*Abyssocladia inflata* Vacelet, 2006	Vacelet, 2006: 573–575, Figs 12-13	Thin pedunculate, flattened discform body, hispid surface, with short filaments	8.5 × 3.5	Axis with longitudinally arranged styles extremely reduced living tissue, body with skeleton of radiating styles protruding from surface	Styles 1075–1800 × 21–33	Undifferentiated	Base missing	Abyssochelae—cleistochelae 80–100 arcuate isochelae 14–150	15–18	Acantho-microxeas 130–350 x 3–5	Easter microplate, East Pacific Rise, bathyal
*Abyssocladia kellyae* Hestetun, Rapp & Pomponi, 2019	Hestetun, Rapp & Pomponi, 2019:5, Fig. 3	Long stalk with disc-shaped body and radial filaments in a single plane on disc margin, enlarged basal plate	43 × 7	Main skeleton with tightly arranged longitudinal mycalostyles, Radiating filaments composed of mycalostyles, subtylostyles and tylostyles within sponge body	Mycalostyles 1262–2321 x19–33	Subtylostyles 1018–1994 x 20–35 tylostyles 275–592 × 6– 15	Undifferentiated	Arcuate isochelae 78– 132	22–32	Absent	Marianas, NW Pacific, abyssal
*Abyssocladia lakwollii* Vacelet & Kelly, 2014	Vacelet & Kelly, 2014:387–392, Fig. 1-3	Pedunculate, flattened disc attached to a thin peduncle with radiating filaments forming a flat to concave umbrella	up to 57	Peduncle tightly packed longitudinal bundles of large mycalostyles, basal attachment tightly packed shorter mycalostyles 1 and substrongyles, mycalostyles 1 of the peduncle fan out in the disc, diverging into radiating bundles towards the rim, disc with many small diverging bundles of mycalostyles 2	Mycalostyles 1, 750–1800 × 15–31, mycalostyles 2, 330–1150 × 6–20	Undifferentiated	Mycalostyles 1, 380–980 × 18–30 substrongyles 250–1150 × 12–30	Anchorate isochelae 1, 110–150 anchorate isochelae 2, 58–92 arcuate isochelae 3, 27–36 cleistochelae 48–70	15–20	Absent	Far eastern Solomon Islands, bathyal
*Abyssocladia marianensis* Hestetun, Rapp & Pomponi, 2019	Hestetun, Rapp & Pomponi, 2019:8, Fig. 4	stalked, long stem with small basal plate attachment, umbrella-shaped body with single horizontal filament crown	130 × 20	stem with tightly packed mycalostyles slightly twisted tracts, umbrella body with radiating bundles of mycalostyles, oxeas in fleshy top part of body	Mycalostyles 1824–2458 × 20–39	Mycalostyles same as stem oxeas 289–477 × 4–7	Undifferentiated	Palmate chelae/ cleistochelae 1, 68–92 palmate chelae/ cleistochelae 2, 26–42	1, 50–65 2, 31–46	Absent	Marianas, NW Pacific, abyssal
*Abyssocladia oxyasters* Ekins, Erpenbeck, Goudie & Hooper 2021	Ekins, Erpenbeck, Goudie & Hooper, 2021:245–251, Figs 4-6	Pedunculate, erect, long thin stem with plano-convex disc shaped body on apex encircled by radiating filaments, and conical basal disc holdfast	140–160 x 0.6	Axis of stem and filaments with longitudinal bundles of mycalostyles 1, basal holdfasts contain smaller thicker curved oxeote anisostyles 2	Mycalostyles 1, 1380–3810 × 15–54	Undifferentiated	Oxeote anisostyles 2, 392–1560 x 20–50	Palmate cleistochelae 62–123 × 17–37	21.5–40 x 2–7	Oxyasters 74-136	Great Australian Bight, bathyal
*Abyssocladia natushimae* Ise & Vacelet, 2010	Ise & Vacelet, 2010:889–892, Figs 2-5	Erect pedunculate long stem on circular base, mop-like inflated apical body with long filaments in one plane, ending with inflated bulbous tips	88 × 1–2.2	Base cored by substrongyles, short microstrongyles and microscleres, axis of peduncle tightly packed with long mycalostyles longitudinally and spirally arranged, upper part of peduncle covered by soft tissue packed with microstrongyles and few microscleres, axis of filaments supported by bundles of mycalostyles and microstrongyles	Mycalostyles 1, 1350 - (1657)-1940 × 19 – (34) -26.5, microstrongyles 14-(64)-250 × 4-(6)-10	Mycalostyles 2, 395 - (1016)-1790 × 10-(16) -24	Substrongyles 395-(642) – 980 × 22 -(36)-45	Cleistochelae-abyssochelae 38-(54)-75	1, 20–23 2, 9–12	Absent	Izu- Ogasawara Arc, Japan, mesophotic
*Abyssocladia polycephalus* Hestetun, Pomponi & Rapp, 2016	Hestetun, Pomponi & Rapp, 2016:523–525, Figs 2-3	Erect pedunculate central stem with side branches each ending in a disclike body bearing filamentous projections	35	Densely packed bundles of mycalostyles in the central stem and branches, radiating bundles of mycalostyles projecting from the body and constituting the skeleton of the filaments, disc- shaped body also with a network of less well organized subtylostyles	Mycalostyles 720–(933)– 1070 × 14– (17)–22	Subtylostyles-mycalostyles 430–(686)– 960 × 5–(10)–13 strongyles 380–(568)– 780 × 15– (18)–22	Base missing	Arcuate isochelae 28– (43)–50	9.4–(9.8)–11.0	Absent	Muir Seamount, Bermuda, bathyal
*Abyssocladia stegosaurensis* Hestetun, Rapp & Pomponi, 2019	Hestetun, Rapp & Pomponi, 2019:11, Fig. 5	Stalked, vertical disc- shaped radial body, filaments radiating in a single plane from disc margin,	48 × 1–3	core axis of longitudinal, tight tracts of mycalostyles, fleshy parts of radial body with subtylostyles and tylostyles bases, base with styles or substrongyles	Mycalostyles 846–1741 x 15–53	Mycalostyles same as stem subtylostyles 491–1134 × 7-17 tylostyles 181–573 × 4-9	Undifferentiated	Palmate isochelae 20–40	6–9	Absent	Marianas, NW Pacific, bathyal

urn:lsid:zoobank.org:act:9CCE875D-9A0A-4513-B95E-50238A9844B0

**Material Examined**. Holotype: WAMZ100664, Cape Range Canyon, site CR7, Ningaloo region, Western Australia, Australia, 21°53′52″S, 112°54′19″E, 2914.1 m, Dive S0338/015, ROV SuBastian, Coll. Wilson, N., Rouse, G., Kirkendale, L. & Ritchie, J. on RV *Falkor*, cruise FK200308, 17 March 2020. 28S GenBank Accession [OR198148].

**Etymology**. Named for after John N. A. Hooper who is a prominent sponge taxonomist and introduced the first author into the wonderful world of sponges.

**Distribution**. This species is presently known only from the type locality in Cape Range Canyon, Ningaloo region, mid Western Australia, from abyssal depths.

**Description**. *Growth form*: The holotype consists of a pedunculated sponge, 47 mm in length, at right angles from a vertical wall, with a slight twist so the disk is facing the ocean surface (Fig. 4A). The sponge has a long stem which supports the centre of the dorsal surface of the disc shaped body. The body has filaments radiating out in a single plane from the disc margin. This is horizontal in the preserved specimen (Fig. 4B), but in a pleasing curve in situ (Fig. 4A). The circular sponge body is 7 mm in diameter, 2 mm thick. There were approximately 40 filaments surrounding the body. The preserved filaments were 7 mm long and 0.1 mm in width, and highly contracted and/or destroyed during collections, compared to the underwater images which show the filaments as up to twice the width of the body (Fig. 4A). The stem length was 43 mm long and 0.4 mm in width.

*Colour*: White underwater, with an orange centre of the body disk, on deck and in ethanol.

*Skeleton*: The thin membranous outer layer of the filaments and sponge body is encrusted with small sigmas and isochelae (Fig. 4C). The stem appears to be devoid of any specific ectosomal layer (Fig. 4D). The axis of the stem, consists of longitudinally arranged styles. The main body and filaments consist of the radiating bundles of subtylostyles and oxeas that form the filaments (Fig. 4C,D).

*Megascleres*: The sponge body and filaments are composed of two different types of spicules. The large mycalostyles are long and straight, (1080-(1367)-1670 x 14.9-(21.6)-33.7 µm, n=41) with a subtle subtylostyle (Fig. 5D,E). Smaller thin and very sharp oxeas (151-(335)-530 x 2.0-(4.0)-6.4 µm, n=27) are also present in the body and filaments (Fig. 5H,I). The stem is composed of a single type of large, slightly sinuous styles (1030-(1630)-2380 x 15.7-(23.7)-31.9 µm, n=23) (Fig. 5F,G). The basal attachment spicules are unknown.

*Microscleres*: The microscleres are composed of a single size class of palmate isochelae (60.3-(88.8)-121.0 x 3.4-(6.7)-9.2 µm, n=27) (Fig. 4A), often with a central saddle (Fig. 5B) and sigmancistras (20.7-(27.8)-32.6 x 1.1-(2.5)-3.2 µm, n=38) (Fig. 5C).

*Molecular data*: The holotype sequenced here produced a sequence very similar to that for *A*. *marianensis* Hestetun, Rapp & Pomponi, 2019 (GenBank MK935689). These two species are nested within one well-supported clade in *Abyssocladia* that contains species from the Great Australian Bight, Macquarie Ridge, Marianas, Solomon Islands and the North Fijian Back Arc Ridge. (Fig. 6).

**Remarks**. This is morphologically closest to one of the specimens of *Abyssocladia jeanvaceleti* and *Abyssocladia stegosaurensis* Hestetun, Rapp & Pomponi, 2019, with the in situ radial filaments. However, the new species differs by having the stem attached to the dorsal face, rather than as a filament as part of the vertical disc as in *A. stegosaurensis. Abyssocladia johnhooperi* sp. nov. also lacks tylostyles, but has oxeas and much larger isochelae.

The centrally-supported disk morphology is closest to *A. oxyasters*, and this is reflected in the phylogenetic placement. This new species differs from *A. oxyasters* by lacking the asters and cleistochelae. *Abyssocladia fryerae* Hestetun, Rapp & Pomponi, 2019, is also another species with a similar disc shaped morphology, but this species from Marianas has very different arcurate isochelae. Another Marianas species i. e. *Abyssocladia marianensis* has a similar in situ morphology but resembles a classic crinorhizoid umbrella shape in preservation, yet the molecular data places these species very close in the phylogeny (Fig. 6). In addition, *A. marianensis* has very different palmate chelae and cleistochelae. This species has similar isochelae to two species recently described^24^ i.e. *Abyssocladia falkor* and *A. jeanvaceleti*, with a similar disc pedunculated shaped body. However, it differs from both by having the disc attached centrally and shaped like a radar dish rather than a flat plane. It also differs markedly from *A. falkor* by the shape of the isochelae and the lack of the protective microspheres and microstrongyles. The new species also differs from *A. jeanvaceleti* by the absence of microstrongyles, the different shaped styles, the absence of saddled isochelae and the presence of the smaller thin oxeas.

### ***Abyssocladia aurora* sp. nov.**

**Fig. 7:**
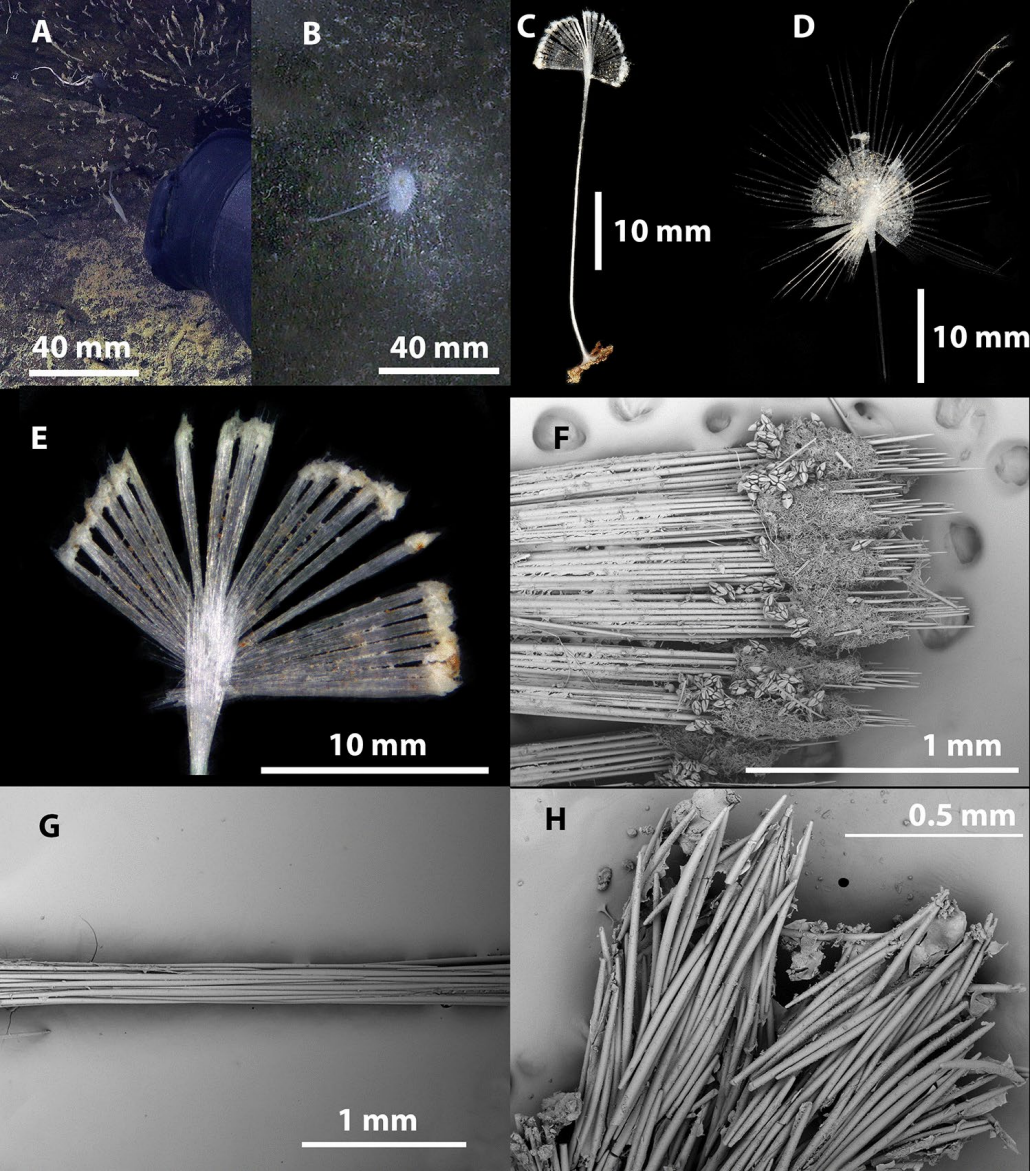
*Abyssocladia aurora* sp. nov. (**A**). Holotype WAMZ100690 in situ before collection. (**B**). Paratype WAMZ100691 in situ before collection. (**C**). Holotype before preservation. (**D**). Paratype WAMZ100691 before preservation. (**E**). Close up of body and filaments of the holotype. (**F**). SEM of the filaments, showing the outer membranous layer pushed along the mycalostyle axis of the filaments. (**G**). Peduncle of the holotype. (**H**). Basal holdfast.

**Fig. 8:**
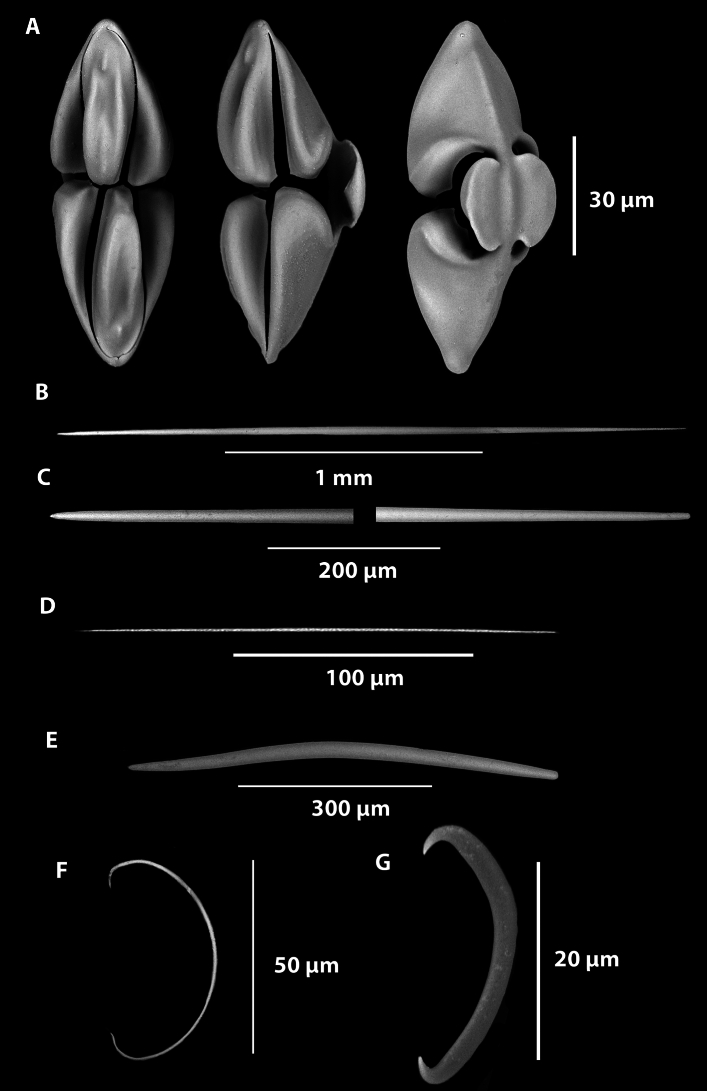
*Abyssocladia aurora* sp. nov. (**A**). Abyssochelae. (**B**). Mycalostyles. (**C**). Magnified ends of the mycalostyle illustrated in (**B**). (**D**). Thin oxetote accessory styles. (**E**). Strongyles in the basal root attachment. (**F**). Thin sigmas. (**G**). Sigmancistras.

Figs. 7, 8, Table 1.

urn:lsid:zoobank.org:act:6823688F-494F-43B3-AAA4-27742FD9A56F

**Material Examined**. Holotype: WAMZ100690, Cape Range Canyon, site CR11, Ningaloo region, Western Australia, Australia, 21°51′ 50”S, 112°41′ 15″ E, 4186.34 m, Dive S0341/020, ROV SuBastian, Coll. Wilson, N., Rouse, G., Kirkendale, L. & Ritchie, J. on RV *Falkor* cruise FK200308, 20 March 2020. Paratype. WAMZ100691, Cape Range Canyon, site CR11, Ningaloo region, Western Australia, Australia, 21°51′ 45″ S, 112°41′ 16″ E, 4355.96 m, dive S0341/008, ROV SuBastian, Coll. Wilson, N., Rouse, G., Kirkendale, L. & Ritchie, J. on RV *Falkor*, cruise FK200308, 20 March 2020.

**Etymology**. Aurora the Roman goddess of dawn.

**Distribution**. This species is presently known only from the type locality in Cape Range Canyon, Ningaloo region, mid Western Australia, from abyssal depths.

**Description**. *Growth form*: Two small pedunculate sponges (Fig. 7A–D), consisting of a thin peduncle bearing a vertically-oriented ovoid body with 54 filaments radiating in a single plane, total length 36 mm. The central body part, where the stem (peduncle) attaches, is only 2 mm wide, 4 mm high and 1 mm thick. The body extends to form a disc of 10-11 mm in width. The filaments extend through this and an extra 15 mm beyond the body in the paratype. The holotype filaments extending outside the body are all missing. The peduncle is 14–28 mm long, and with a basal holdfast on the holotype.

*Colour*: White underwater on deck and in ethanol.

*Skeleton*: Thin membranous layer of the filaments and sponge body is encrusted with small sigmas and abyssochelae (Fig. 7F). The stem and basal attachment appear to be devoid of any specific ectosomal layer (Fig. 7G,H). The axis of the stem consists of longitudinally-arranged mycalostyles. The main body and filaments also consist of the radiating bundles of mycalostyles that form the filaments (Fig. 7E,F). There are rare, small and thin accessory styles.

*Megascleres*: The sponge body, filaments and peduncle are composed of large mycalostyles. For the holotype, the large mycalostyles are long and straight (936-(1707)-2450 x 14.9-(24.4)-38.1 µm, n=52) and the rare thin accessory styles (98.2-(163.4)-218.0 x 1.9-(2.5)-2.8 µm, n=5). For the paratype, the mycalostyles (830-(2006)-2530 x 19.1-(26.6)-37.8 µm, n=34) and accessory styles (147-(198)-267 x 1.3-(3.0)-3.8 µm, n=15) (Fig. 8B–D). The basal attachment spicules are strongyles (471-(694)-1030 x 20.3-(27.0)-36.2 µm, n=38) for the holotype.

*Microscleres*: The microscleres are composed of a single size class of abyssochelae (length 47.4-(77.7)-142.0 x maximum width 24.7-(32.7)-50.7 µm, n=57) (Fig. 8A), often with a central saddle (Fig. 8A). The abyssochelae for the paratype were similar (65.7-(99.2)-177 x maximum width (26.8-(37.8)-62.5 µm, n=11). There were also large thin sigmas (29.9-(43.7)-55.3 x 0.6-(1.0)-1.6 µm, n=24), (Fig. 8F) and sigmancistras (21.2-(25.8)-30.7 x 1.3-(2.2)-3.1 µm, n=50) (Fig. 8G). The large thin sigmas were rare in the paratype (50.8-(55.4)-60.0 x 1.7-(1.8)-2.0 µm, n=2), but the sigmancistras were common (22.3-(25.9)-30.1) x 1.7-(2.5)-4.3 µm, n=21).

**Remarks**. This new species bears a similar resemblance to those with vertically orientated discs such as *Abyssocladia dominalba* Vacelet, 2006*, A. natushimae* Ise & Vacelet, 2010 and *Patriciacladia enigmatica* Kelly et al. 2023. However, it is clearly different from *A. natsushimae* Ise & Vacelet, 2010 as it lacks the microstrongyles, has different shaped abyssochelae and has large thin sigmas. It is also different from *A. dominalba* as it lacks the anisochelae and the acurate isochealae. *Patriciacladia enigmatica* is in a different genus because of the palmate isochelae and anisochelae. *Abyssocladia huitzilopochtli* Vacelet 2006, has very different abyssochelae. This species differs from *A. bruuni* in the shape of the abyssochale and the presence of thin sigmas. It differs from *A. claviformis* Koltun, 1970 by the shape of the abyssochleae the lack of tylostyles and in the new species the presence of thin sigmas. The abyssochelae of *A. aurora* sp. nov. resemble in shape those of *A. escheri* Ekins, Erpenbeck & Hooper 2020 from the east coast of Australia and *A. natsushimae* from the North Pacific. However the abyssochelae in this new species (47.4-(77.7)-142.0 µm) are larger than those of *A. escheri* (18.4-(29.3)-53.1 µm) and *A. natsushimae* (38-(54)-75 µm), and additionally *Abyssocladia aurora* sp. nov. lacks the microstrongyles that are present in *A. natsushimae*.

### ***Abyssocladia janusi* sp. nov.**

**Fig. 9:**
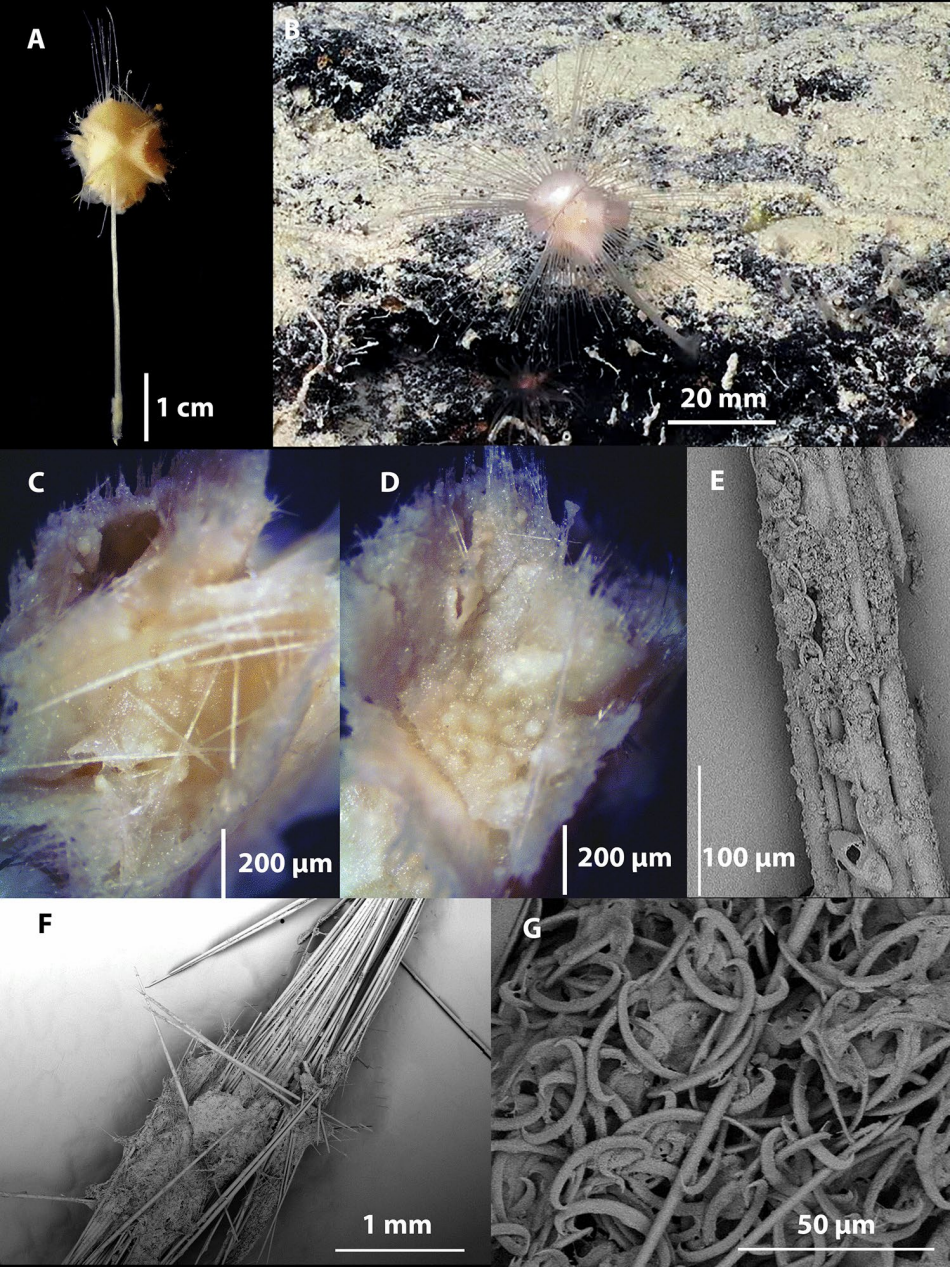
*Abyssocladia janusi* sp. nov. (**A**). Holotype WAMZ100501 before preservation. (**B**) Holotype WAMZ100501 in situ before collection. (**C**). Closeup of the body showing two faces. (**D**). Looking into one of the faces to see embryos. (**E**). SEM of a filament, showing longitudinal mycalostyles and chelae and sigmancistras of the outer layer. (**F**). The mycalostyles making up the palisade walls of the faces, and the outer layer of sigmas and small styles. (**G**). Closeup of the encrusting sigmas and thin styles.

**Fig. 10:**
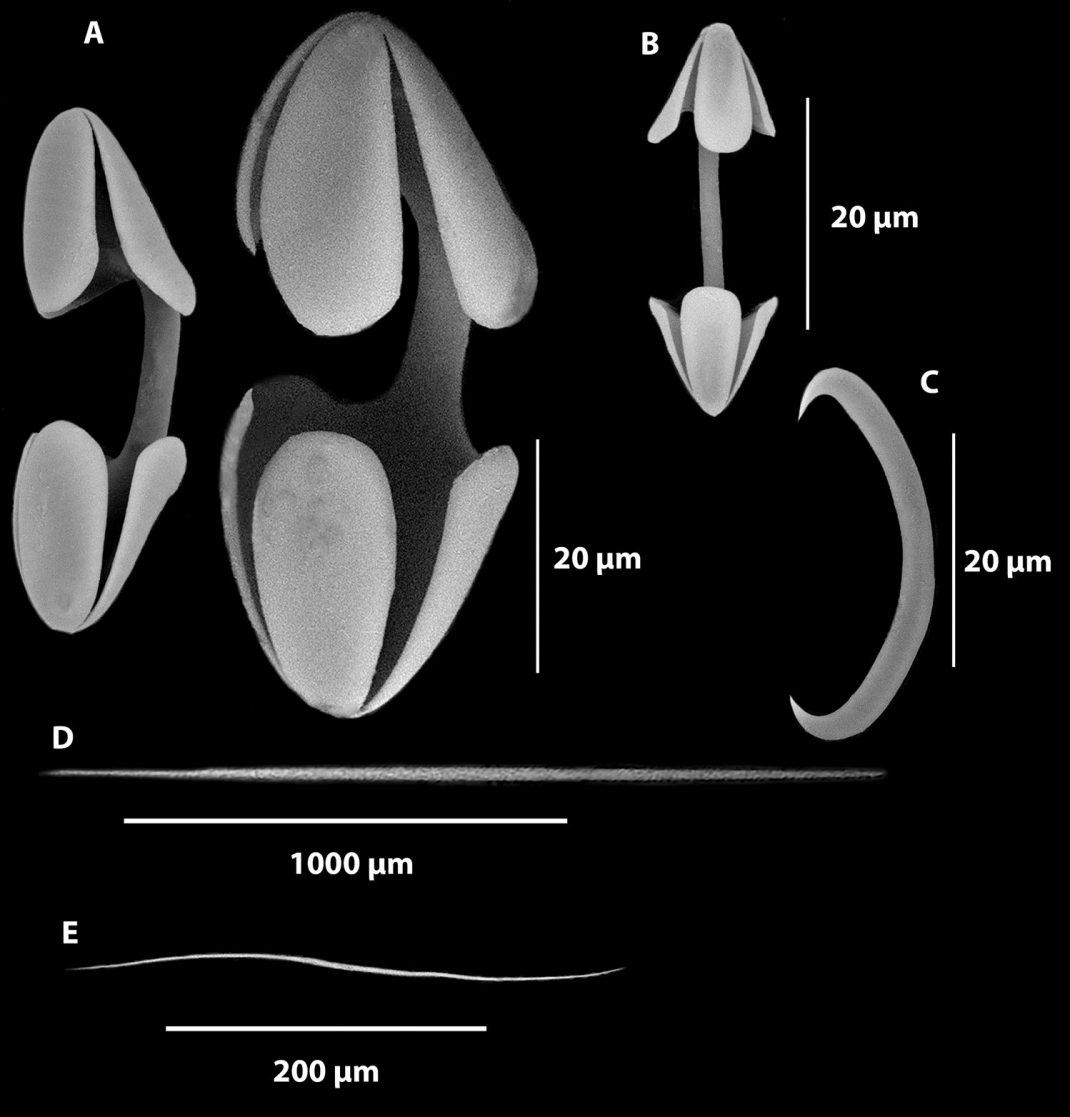
*Abyssocladia janusi* sp. nov. (**A**). Large isochelae. (**B**). Small Isochelae. (**C**). Sigmancistra. (**D**). Mycalostyles. (**E**). Smaller sinuous oxeas.

Figs. 9,10, Table 1.

urn:lsid:zoobank.org:act:9652872D-528A-47DA-979B-9EE53BBD2BF0

**Material Examined**. Holotype: WAMZ105001, Bremer Canyon system, Knob C, Site FK20_312_10B, Western Australia, Australia, 35°3′ 16″ S, 119°44′ 30″ E, 3302.21 m, Dive S0312, ROV *SuBastian*, Coll. Hosie, A.M & Hara, A. on RV *Falkor,* cruise FK200126, 28 January 2020.

**Etymology**. Named after the Roman God Janus, who has two or four faces, in recognition of the many faces of the sponge body.

**Distribution**. This species is currently known from the type locality Knob C in the Bremer Canyon system, south Western Australia, Australia, from abyssal depths.

**Description**. *Growth form*: The holotype consists of a small pedunculate sponge (Fig. 9A–C), consisting of a thin peduncle bearing a subspherical body with seven faces. Each of the faces is conical in shape and at the bottom of the cone are the embyros, resembling a bird’s nest full of eggs (Fig. 9B,C). Between the faces were found amphipods, presumably in the process of being digested (Fig. 9D,E). The body was 20 x 20 x 8 mm, flattened perhaps by preservation. The total length of the sponge was 55 mmm. The peduncle was 42 mm in length and 2 mm in diameter. In the preserved holotype there are scattered fragments of filaments, from the in situ images the filaments form an extension of the palisade around each face forming a graceful cone extending from each face. The filaments are broken, the largest intact filament was 20 mm in length, judging from the in situ images they are estimated at 30 mm in length.

*Colour*: Cream on deck and in ethanol.

*Skeleton*: Thin membranous layer of the sponge body is thickest around the reproductive structures, where it is mainly proteinaceous. Where the outer layer is directly covering the mycalostyles with a thin membrane it contains the small oxeas, the rare chelae and the ubiquitous sigmas (Fig. 9E,G). The stem and basal attachment appear to be devoid of any specific outer layer. The axis of the stem, consists of longitudinally arranged styles. The main body and filaments consists of the radiating palisade of styles that form the conical faces (Fig. 9B,D).

*Megascleres:* The sponge body, filaments and peduncle are composed of a single large oxetote mycalostyles. The large mycalostyles are long and straight (1730-(2107)-2390 x 21.5-(35.0)-50.4, µm, n=23) (Fig. 10D. Smaller, very thin, sharp, sinuous oxeas (372-(526)-643 x 3.4-(5.8)-8.7 µm, n=28) are also present in the body (Fig. 10E).

*Microscleres:* The microscleres are composed of two size class of palmate isochelae. The largest measures 45.4-(60.7)-74.6 (maximum length) x 16-(24.)-35 (maximum width) x 2.7-(3.7)-5.2 µm (stem width), n=13) (Fig. 10A). The smaller palmate isochelae measures (24.7-(29.7)-36.3 (maximum length) x 7.4-(9.3)-12.0 (maximum width) x 1.0-(1.4)-2.1 µm (stem width), n=18 (Fig. 10B). Sigmancistras are also present, that have a slight twist of less than 30°, they measure (28.1-(32.5)-37.5 x 1.5-(2.8)-3.9 µm, n=31) (Fig. 10C).

**Remarks**: This new species of *Abyssocladia* is unique in its formation, with the multiple nest-like faces on the sponge body. This is perhaps some modification to increase its reproductive success, by ensuring different trajectories for embryo release. This new *Abyssocladia* is similar to many species with isochelae, mycalostyles and sigmancistras as a spicule composition. However, the only *Abyssocladia* species with palmate isochelae are *A. johnhooperi* nov. sp., *A. jeanvaceleti* and *A. stegosaurensis*. The new species can be distinguished from *A. johnhooperi* nov. sp. by the morphology, the very two different size and shapes of the palmate isochelae, and the lack of subtyle development of the mycalostyles. *Abyssocladia jeanvaceleti* also has different morphology, subtylostyles and many different shaped and sized mycalostyles and lacks the sinuous oxeas in the new species. *Abyssocladia stegosaurensis* has several tylostyles and subtylostyles, much smaller (and differently shaped) anisochelae and smaller sigmancistras. The nest-like formation (Fig. 9A) resembles the sheltered embryos in *Asbestopluma* (*Helophloeina*) *formosa* Vacelet 2006, but these two are in entirely different genera.

### Genus *Axoniderma* Ridley & Dendy, 1886

**Diagnosis**. Cladorhizidae with anchorate anisochelae and a crinorhizoid parasol morphology.

**Type species**. *Axoniderma mirabilis* (Ridley & Dendy, 1886)

**Remarks**. There are nine currently accepted species in *Axoniderma*. All species are listed and compared in Table 2. *Axoniderma* species containing structures emerging from the body disc were recently compared^24^.

**Table 2:** A comparison of all *Axoniderma* species.

Species	Source	Morphology	Total height x stem width (mm)	Skeleton	Spicules of main axis (LxW um)	Spicules of lateral filaments or body (LxW um)	Spicules of basal attachment (LxW um)	Chelae (L um)	Sigmancistras (L um)	Sigmas (L um) and other microscleres	Locality/ depth range
*Axoniderma challengeri* sp. nov	This work	Pedunculated sponge, with a long thin stem with a hemispherical sponge body, showing the classic ‘cladorhizid’ shape	75 × 0.3	Axis of stem and body cored by longitudinal bundles of mycalostyles	Oxeotote mycalostyle 454–3340 × 4–54	Oxeotote mycalostyle 454–3340 × 4–54	Oxeotote mycalostyle 454–3340 × 4–54	Quadradentate anchorate unguiferate anisochelae 23–30 × 2–4	Sigmancistras 44–54 × 1–4	Absent	Ningaloo, Western Australia, abyssal
*Axoniderma australis* (Ekins, Erpenbeck & Hooper, 2020)	Ekins, Erpenbeck & Hooper, 2020a:47–72, Figs 7-8	Pedunculate erect, unbranched, ‘crinorhizoid’ parasol- shaped sponge with filaments nearly horizontal to the disc- shaped body, long thin stem, basal attachment missing	75 × 2	Axis of stem and body cored by longitudinal bundles of mycalostyles of 3 size/ shape classes	Mycalostyles 1, 1380–4960 × 21–85, mycalostyles 2, 1000–1940 × 15–30, mycalostyles 3, 485–1080 × 15–30	Undifferentiated	Undifferentiated	Unguiferate anisochelae 24–35 × 2–5 cleistochelate anisochelae 35–44 × 18–32	Absent	Sigmas 43–100 × 2–5	East Australia,off Tasmania and New South Wales, abyssal
*Axoniderma corona* (Lehnert, Watling & Stone, 2005)	Lehnert, Watling & Stone, 2005:1359, Figs 1-2	Pedunculate erect, parasol ‘crinorhizoid’ morphology, long stem, two planes of different appendages, basal disk holdfast	225–325 × 1–9	Axis of stem and basal appendages with thick bundles of long mycalostyles, crown with tracts of long mycalostyles fanning out in 1 plane, with single thin subtylostyles perpendicular to the axial skeleton, basal plate with mycalostyles and short anisoxeas densely packed in one plane parallel to the substrate	Mycalostyles 600–4260 × 10–65	(Sub)tylostyles 510–1650 × 8–20 (in the crown)	Anisoxeas 140–660 × 38–43	Anchorate anisochelae 30–42	35–42	Absent	Aleutian Islands, mesophotic-bathyal
*Axoniderma hubbsi* (Lundsten, Reiswig & Austin, 2017)	Lundsten, Reiswig & Austin, 2017:255–257, Fig. 6	Erect parasol morphology, with ‘chrinorhizoid’ filaments at base of conical body, apex flat, on thin stalk, basal attachment missing	110 × 1.1	3 size classes of mycalostyles, presumed undifferentiated distribution in sponge	Mycalostyles 1, 4298–5836 × 60-80, mycalostyles 2, 3712–4632 × 43–79, mycalostyles 3, 2097–3033 × 29–59	Undifferentiated	Undifferentiated	Tridentate unguiferate anisochelae 29–33	85–103	Pseudoamphiasters 128–144	California, USA, bathyal
*Axoniderma kensmithi* (Lundsten, Reiswig & Austin, 2017)	Lundsten, Reiswig & Austin, 2017:250–253, Figs 2-3	Crinorhizoid’ form with numerous filaments parasol-shaped, on long stem with densely branching basal rhizoid holdfast, 2–4 spermatocyst-bearing discs on short, slender stalks on apex of body	208–325 × 1–2.1	Axis of stem cored by mycalostyles 1–3, filaments cored by mycalostyles 1&3, basal rhizoid cored by strongyles and mycalostyles 3	Mycalostyles 1, 3138–4850 × 49–75, mycalostyles 2, 1009–1501 × 15–29, mycalostyles 3, 242–452 × 13–17	Mycalostyles 1 & 3	Strongyles 421–1023 × 10–22 mycalostyles 3	Tridentate unguiferate anisochelae 33–37	43–50	Absent	West Coast, USA, bathyal-abyssal
*Axoniderma longipinna* (Ridley & Dendy, 1886)	Ridley & Dendy, 1887:92, P1. XX. Fig. 2; P1. XXI. Figs 4, 21; Koltun 1970: 185–186, Fig 13 PL VI Figs 3-5	Pedunculate erect, ‘crinorhizoid’ parasol morphology, long thin stem with apical suspherical body bearing long radial projections outwards and downwards, basal attachment not recorded	27 × 5	Axes of stem and lateral processes cored by bundles of long mycalostyles	Mycalostyles 1, 3000 × 50–55 mycalostyles 2, 200–1000 × 10–16	Undifferentiated	not recorded	Unguiferate tridentate anisochelae 30–78	Absent	Absent	NW Pacific, bathyal—hadal
*Axoniderma mirabilis* (Ridley & Dendy, 1886)	Ridley & Dendy, 1887:98, Pl XX, Fig. 5, Pl. XXI, Figs 8-10; (Ekins, Erpenbeck & Hooper 2020a:76–78, Fig. 11); [Koltun 1970:187, Fig. 13	Pedunculate erect, ‘crinorhizoid’ parasol- shaped with conical cap- shaped body with terminal papillae, perched on the end of a long slender stem, with numerous long filaments surrounding the body, basal attachment not recorded	56 ? (13 × 2)	Axis of stem, body and filaments cored by bundles of styles- mycalostyles of a single size class, with single styles projecting on the terminal papillae, cortex encrusted with pseudoamphiasters and other microscleres	Styles > 3500 (styles- mycalostyles 1, ~ 6000 × 11, styles 2, ~ 300 × 5) [mycalostyles ~ 3500 × 15–50]	Undifferentiated	Absent	Tridentate unguiferate anisochelae 38 (31–48)[‘anchors’ 33–38]	Absent	Sigmas 75.6 (88–114) [sigmas 76] birotules (‘amphiasters’) with 5 terminal alae 230 (pseudoamphiasters 127–295) [‘amphiasters’ 88–230]	SE Pacific, off S Easter Island, & N Pacific, abyssal
*Axoniderma mexicana* (Lundsten, Reiswig & Austin, 2017)	Lundsten, Reiswig & Austin, 2017:253, Figs 4-5	Erect parasol-shaped, ‘crinorhizoid’ body with radiating long filaments at base of body, on long stalk, presumed basal holdfast not collected	300 × 1–3	3 size classes of mycalostyles, presumed undifferentiated distribution in sponge	Mycalostyles 1, 3577–4133 × 58–74, mycalostyles 2, 2650–3246 × 45–63, mycalostyles 3, 1293–1931 × 23–39	Undifferentiated	Undifferentiated	Tridentate unguiferate anisochelae 33–36	47–55	Asymmetrical pseudoamphiasters 102–115	West coast USA, Mexico, bathyal
*Axoniderma poritea* (Ekins, Erpenbeck & Hooper, 2020)	Ekins, Erpenbeck & Hooper 2020a:72–75, Figs 9-10	Pedunculate erect, unbranched, ‘crinorhizoid’ parasol- shaped sponge with conical acuminate body with filaments extending downwards, long thin stem, basal attachment missing	10–50 × 1–1.5	Axis of stem and body cored by longitudinal bundles of mycalostyles with variable size range, filaments 10–30 mm consisting of smaller bundles of mycalostyles	Mycalostyles 523–6650 × 8–75	Undifferentiated	Unknown	Unguiferate anisochelae 20–36 × 1.6–4.2	Absent	Asymmetrical pseudoamphiasters 93–168 × 6–15.3	Off Fraser Island, Queensland, and Freycinet Peninsula, Tasmania, abyssal
*Axoniderma similis* (Ridley & Dendy, 1886)	Ridley & Dendy, 1886:343;Ridley & Dendy, 1887:93, Pl. XX, Fig. 7, Pl. XXI, Fig. 5, 18; (Lévi, 1993: 40, Fig. 14, Pl. V. Figs 4-6); [Ekins, Erpenbeck & Hooper 2020a:85–86, Fig. 16]	Pedunculate erect, ‘chrinorhizoid’ parasol morphology, with cap shaped conical body and long filaments encircling the lower body, perched on thin stem,basal attachment missing	?	Axis of stem, body and filaments cored by bundles of styles, dense close-set tylostyles mostly near surface, fewer in the axial skeleton	Styles 'very long and slender’ tylostyles 210–600 × 16(styles 1, up to 3600 × 50, styles 2, 650–700 × 30, styles 3, 250–300 × 8–10) [Myclostyles large 1950–3550 × 18–30, subtylostyles 480–810 × 12–24, small subtylostyles 194–260 × 12–20]	Undifferentiated	Undifferentiated	Tridentate unguiferous anisochelae 30 (anisochelae 30)[24-30]	Absent	Absent	Central Pacific & New Caledonia, bathyal-abyssal
*Axoniderma wanda* Ekins & Hooper 2023	Ekins & Hooper, 2023:459–468, Figs 11-12	Pedunculate erect, unbranched long thin stem with a disc shaped body with radial filaments and on a long stalk a circular terminal lure	60 × 0.5	Axis of stem and body cored by longitudinal bundles of mycalostyles of 2 size classes and an extra thin small style located in the lure	Mycalostyles 1, 903–2360 × 24–56, mycalostyles 2, 420–1090 × 7–24	Lure styles 260–320 × 3–11	Strongyles 280–1150 × 15–38	Tridentate unguiferate anisochelae 22–29 × 1–3	Sigmancistras 20–36 × 1–4	Absent	Great Barrier Reef, Australia, bathyal

### *Axoniderma challengeri* sp. nov

**Fig. 11:**
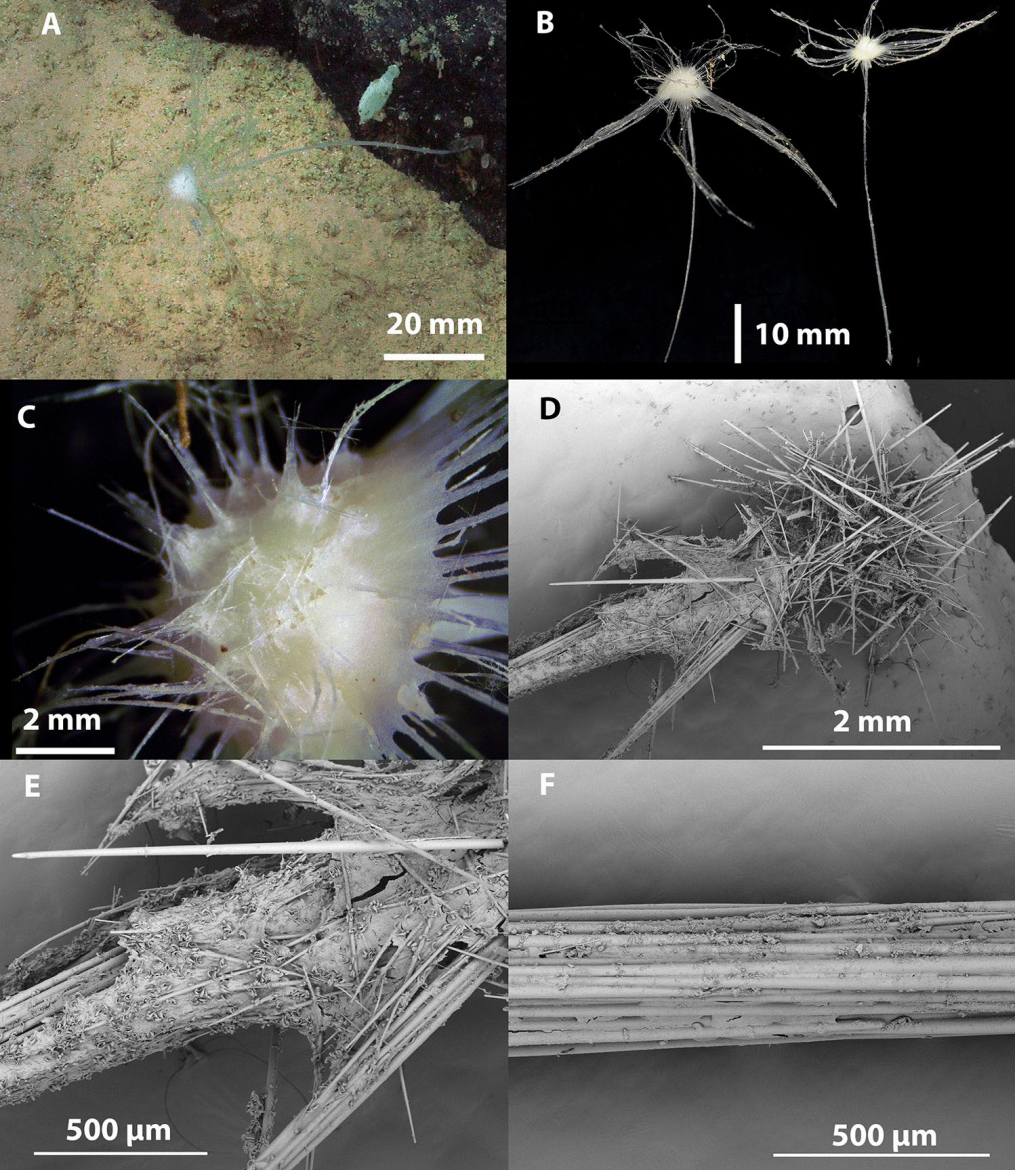
*Axoniderma challengeri* nov. sp. (**A**). Holotype WAMZ100734 in situ before collection. (**B**). Holotype WAMZ100734 on the left and paratype WAMZ100736 on the right before preservation. (**C**). Sponge body showing radiating filaments. (**D**). Filaments emerging from exposed skeleton of the sponge body. (**E**). Surface of the filaments and sponge body, with the axis of both also showing (**F**). Membranous outer layer with the underlying mycalostyle axis of the peduncle of the sponge.

**Fig. 12:**
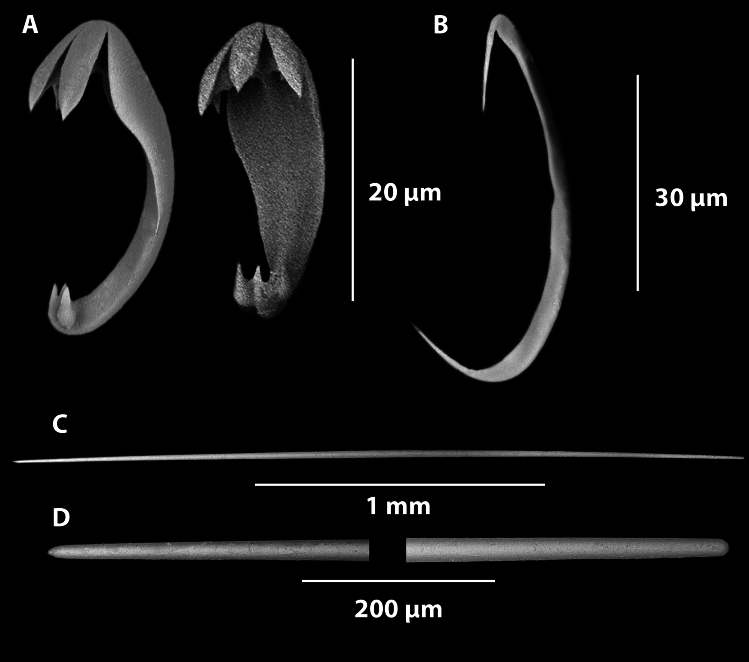
*Axoniderma challengeri* sp. nov. (**A**). Anisochelae. (**B**). Sigmancistra. (**C**). Mycalostyle. (**D**). Magnified ends of the mycalostyle illustrated in (**C**).

Figs. 11, 12, Table 2.

urn:lsid:zoobank.org:act:511CA3DB-BF73-48DC-99CB-D61F56E5D710

**Material examined**. Holotype: WAMZ100734, Cape Range Canyon, site CR13, Ningaloo region, Western Australia, Australia, 21°49′ 4″ S, 112°30′ 39″ E, 4514.2 m, Dive S0347/013, Coll. Wilson, N., Rouse, G., Kirkendale, L. & Ritchie, J. on RV *Falkor* cruise FK200308, 27 March 2020. Paratype: WAMZ100736, same collection details as the holotype, same rock.

**Etymology**. This species is named for its resemblance to *Axoniderma longipinna* (Ridley & Dendy 1886) described from the Challenger expedition.

**Distribution**. This species is presently known only from the type locality in Cape Range Canyon, Ningaloo region, mid Western Australia, from abyssal depths.

**Description**. *Growth form*: The holotype consists of a pedunculated sponge, with a long thin stem that terminates in a hemispherical sponge body, showing the classic cladorhizid shape. The body is almost hemispherical, facing upwards from the peduncle. In life both sponges were emerging sideways from the same rock with the face turned mostly upwards towards the ocean surface (Fig. 11A). The total length of the holotype is 75 mm, and the paratype is 65 mm in length. With the peduncle 55 mm in length and 0.33 mm in width in both specimens. There are 35-40 remaining long radiating filaments from the circumference of the body up to 31 mm in length (Fig. 11B). There are also shorter filaments rising from the top hemispherical part of the sponge body at regular intervals (Fig. 11C). The sponge body diameter is 10 mm and 6 mm high in the holotype and 5 mm in diameter in the paratype. The filaments are 31 to 23 mm long and 0.15 to 0.25 mm in width.

*Colour*: The sponge body, filaments and stem are all off white in life and in ethanol preservative.

*Skeleton*: The body, the stem and the filaments are covered on the external surface with a thin membranous layer up to 50 µm thick containing tridentate unguiferate anisochelae and sigmancistras, (Fig. 12A,B). The skeleton of the body, the filaments, the stem and the basal attachment consist of mycalostyles in concentrated longitudinal bundles (Fig. 12E,F).

*Megascleres*: The megascleres consist of a single type of oxeotote mycalostyle with a blunt termination (Fig. 12C,D). Initial observations looked like there were two sizes, but because of the continuous size ranges and the lack of shape differentiation or localisation of the styles, it indicates they are merely size differences throughout the sponge. The styles are (454-(1577)-3340 x 4.0-(26.4)-53.7 µm, n=89) in the holotype and (439-(1865)-2640 x 8.4-(35.8)-54.9 µm, n=47) in the paratype.

*Microscleres*: The microscleres consist of abundant small anchorate unguiferate anisochelae with three large alae and three smaller alae on each end (Fig. 12A). They are (22.8-(27.2)-30.4 x 1.8-(2.7)-3.5 µm, n=45) in the holotype and (22.7-(27.0)-30.6 x 2.5-(3.5)-4.9 µm, n=39) in the paratype. Sigmancistras are rare, contort to almost 90° and consist of a single size class (Fig. 12B). They are (50.5-(51.9)-54 x 1.4-(2.2)-3.2 µm, n=3) in the holotype and (43.9-(45.1)-47.5 x 3.1-(3.1)-3.1 µm, n=3) in the paratype.

**Remarks**. This species has the unguiferate anchorate anisochelae and sigmancistras common to many other species of *Cladorhiza, Axoniderma, Bathytentacular* and *Nullarbora.* All species previously and currently belonging to *Cladorhiza* were compared in Table 5^5^ and grouped morphologically by a visual icon. Morphologically, this new species has a cladorhizid parasol shape with additional small filaments radiating upwards from the body, and it resembles *Axoniderma mirabile* (Ridley & Dendy 1886), however it lacks the characteristic pseudoamphisters. The appearance of this new species also resembles *A. longipinna* (Ridley & Dendy 1886), and the differences are discussed below. The new species *Axoniderma challengeri* nov. sp., has a similar basic morphological shape and ancorate anisochelae to *A. corona* (Lehnert, Watling & Stone, 2005), *A. kensmithi* (Lundsten, Reiswig & Austin, 2017) and *A. mexicana* (Lundsten, Reiswig & Austin, 2017). *Axoniderma mexicana* has the presence of pseudoamphiasters, a trait shared with *A. mirabile* and *A. poritea* (Ekins, Erpenbeck & Hooper, 2020). *Axoniderma kensmithi* has two to four disc-like lures. The recently described species from eastern Australia, *A. wanda*, has an obvious long lure. *Axoniderma corona* has a large crown-like lure emerging from the centre of the disc.

*Axoniderma longipinna* is the only other sponge with a disc and some extra structures displaying the classic crinorhizoid parasol morphology. In the description^3^, a second circle of short processes around the summit is mentioned, similar to that in *A. mexicana*, another North Pacific species. In the description of *A. longipinna* the anisochelae are 34–60 µm, the larger ones located on the lower surface of the sponge. The styles are fusiform 3000 x 50 µm and 1000 x 15 µm, with two or possibly three size classes. In the description the sponge has about 25 to 30 processes being 19 mm in length.

Koltun^25^ recorded *A. longipinna* and *A. similis* (Ridley & Dendy, 1886), the latter which he considered a synonym, when he based his descriptions on six specimens from the in the Vityaz collection, from the Northern Pacific. Unfortunately, because he combined both species, it is difficult to determine which spicules belonged to each species. The three-pronged ansiochelae were described by Koltun^25^ as often belonging to two size classes, i.e. large and small, and he gave a measurement of 0.030-0.078 mm long. Re-examination of the type of *A. similis* in^5^, showed the anisochelae measurements to be 23.5-(26.7)-29.8 µm, by process of deduction, then the other ansiochelae measured by Koltun^25^ is about 70 µm. However, Ridley & Dendy^3^ also talk about the size difference in anisochelae, with larger ones occurring on the lower surface of the sponge.

*Axoniderma longipinna* occurring in the North Pacific, is clearly the most similar sponge. However, this new species differs from *A. longipinna* by the far greater number of radial filaments, the hemispherical shape of the upper surface of the sponge body having radial emergence of filaments in all directions, and not just a second circle of filaments, the presence (albeit rare) of sigmancistras and the broader upper alae of the small anisochelae. To date, there is not available molecular data to assist testing this hypothesis.

### Genus Cladorhiza Sars, 1872

*Trochoderma* Ridley & Dendy, 1886: 344

*Axoniderma* Ridley & Dendy, 1886: 493

*Exaxinata* de Laubenfels, 1936: 122

*Raoa* de Laubenfels, 1936: 123

**Definition**. Cladorhizidae with anchorate anisochelae and arbuscular morphology.

**Type species**. *Cladorhiza abyssicola* Sars, 1872 (by monotypy)

**Remarks**. There are currently at least 25 accepted species in *Cladorhiza*. All known arborescent species of *Cladorhiza* are listed in Table 3.

**Table 3:** Comparative morphological and distributional data for all known arborescent species of *Cladorhiza*.

Species	Source	Morphology	Total height x stem width (mm)	Skeleton	Spicules of main axis (LxW um)	Spicules of lateral filaments or body (LxW um)	Spicules of basal attachment (LxW um)	Chelae (L um)	Sigmancistras (L um)	Sigmas (L um) and other microscleres	Locality/ depth range
*Cladorhiza vanessaekins* sp. nov	This work	Arbuscular- arborescent erect, tree-like branched central stem with numerous side branches in several planes, each branch bearing numerous thin filaments and terminating in a small swelling, basal stem anchored by holdfast	67- 26 × 1	Axis, branches and filaments all consist of a dense core of longitudinally arranged mycalostyles mycalostyles of filaments anchored into main axial skeleton	Mycalostyles 556-(864)-2170 × 7.4-(21.4)-44.9	Undifferentiated	Undifferentiated	Anchorate anisochelae 22.00-(27.4)-33.7	Absent	Sigmas 66–168	Western Australia, abyssal
*Cladorhiza abyssicola* Sars, 1872	Lundbeck 1905:79; Sars, 1872:65-6, Pl. VI, Figs 16-348; Hestetun, Fourt, Vacelet, Boury- Esnault & Rapp, 2015: 1330–1332, Fig. 12; Hestetun, Tompkins- Macdonald & Rapp, 2017b:22, Figs 16-17	Arbuscular- arborescent erect, tree-like branched central stem with numerous side branches in several planes, each branch bearing numerous thin filaments and terminating in a small swelling, basal stem anchored by rhizoids	15–45 × 1–2	Axis, branches and filaments all consist of a dense core of longitudinally arranged mycalostyles/strongyles, mycalostyles of filaments anchored into main axial skeleton	Mycalostyles- strongyles 270-(486)-930 × 5.2-(12.6)- 25.1	Undifferentiated	Undifferentiated	Anchorate anisochelae 15.0-(20.5)-28.6	31.4-(40.4)- 52.8	Sigmas 1, 76–140 sigmas 2, 30–45	North and Mid- Atlantic, Mediterranean, North Sea and Arctic, mesophotic- bathyal
*Cladorhiza corallophila* Göcke, Hestetun, Uhlir, Freiwald, Beuck & Janussen, 2016	Göcke, Hestetun, Uhlir, Freiwald, Beuck & Janussen, 2016:514, Figs 2-4	Arbuscular erect, multiple stems producing bushy colonies, lateral processes project from stems in 1 plane, covered in filaments, no distinct basal attachments	100 × 2	Axis of stems and branches cored by dense aggregation of mycalostyles, strongyles and occasional oxeas, filaments with few megascleres	Mycalostyles 280–420 x 8–14.4 strongyles 200–410 x11.2–17.6 oxeas 300–440 x 6.4–9.6	Undifferentiated	Undifferentiated	Anchorate unguiferous anisochelae 17.6–24	Absent	Sigmas 75.2–88	Mauritania, mesophotic
*Cladorhiza corticocancellata* Carter, 1876	Carter, 1876:27; Hestetun, Tompkins- Macdonald & Rapp, 2017:25–27, Figs19–20	Arbuscular- arborescent erect, tree- like, dichotomously branched, stem covered in a large number of partly coalesced filaments with numerous cavities, connected to by an amorphous growth or plate	200 x ?	Axis of stem with bundles of mycalostyles forming a solid core, axis of filaments made up of tightly packed mycalostyles perpendicular to the centre of the main skeleton	Mycalostyles 475-(670)-799 × 9.4-(17.5)- 25.1	Undifferentiated	Undifferentiated	Anchorate anisochelae 25.3-(32.7)-37.7	46.6–61.3-95.5	112–155-182	Shetland and NE Atlantic, bathyal
*Cladorhiza flosabyssi *Topsent, 1909	Topsent, 1909:12; Lehnert al. 2005: 1361	Arbuscular erect, long thin stem, long thin flower-like bouquet of filaments at apex of stem, no basal attachment	115 x ?	Axis of stem and filaments cored by longitudinal bundles of mycalostyles, surrounded by tylostyles in the flesh	Mycalostyles 3000–5000 × 70 tylostyles 400–700 x 4–12	Undifferentiated	Undifferentiated	Arcuate unquiferate anisochelae 24–1	Absent	Sigmas 42–47	Central Atlantic, Cape Verde, bathyal
*Cladorhiza gelida* Lundbeck, 1905	Lundbeck, 1905:83, Pl.3 Fig. 1, Pl. 11 Fig. 3; Hestetun, Fourt, Vacelet, Boury- Esnault & Rapp 2015:1334–1335, Fig. 14; Koltun 1959:81–82; Hestetun, Tompkins- Macdonald & Rapp 2017:27–30, Figs 21–22	Arbuscular erect, branched tree- like with one or several main stems with numerous pinnate branches in 1 plane, branches covered in numerous filaments, short stem with basal plate or rootlets	250 × 2–4	Axis of stem, root and body with bundles of subtylostyles- mycalostyles, without differential distribution in the sponge	Subtylo to mycalostyles 380-(622)- 1000 × 7.0- (16.6)-25.1	Undifferentiated	Undifferentiated	Ancorate anisochelae 22.8-(31.2)-42.3	37.7–52.3-85.8	Sigmas 86-(139)- 174	North Atlantic, bathyal-abyssal
*Cladorhiza iniquidentata* Lundbeck, 1905	Lundbeck, 1905:91–92, Fig. 4, Pl. III, Fig. 6, Pl. XII, Fig. 5; Hestetun, Tompkins- Macdonald & Rapp, 2017:30	Arbuscular erect, with central stem and side branches covered in filaments projecting in all directions	43 × 1–2	Axis of stem and branches with longitudinal bundles of mycalostyles, axis of filaments with overlapping mycalostyles anchored into the main stem	Mycalostyles 487-(600)-688 × 18.9-(22.4)-26.2	Undifferentiated	Undifferentiated	Anchorate anisochelae 19.6-(23.6)-28.8	Absent	Absent	Iceland-Faroe Ridge, bathyal
*Cladorhiza methanophila* Vacelet & Boury-Esnault, 2002	Vacelet & Boury-Esnault, 2002:142; Hestetun, Fourt, Vacelet, Boury- Esnault & Rapp, 2015: 1335	Arbuscular erect, branched tree- like, central stem and branches covered with numerous unbranched filaments, basal disc holdfast	44–400 x 5–6	Axis of stem and branches with bundles of mycalostyles, spirally twisted in the main axis, filaments with fewer mycalostyles embedded in and projecting from axis, basal disc with thicker mycalostyles	Mycalostyles 310–960 x 5–20 styles 160–320 x 1.6–4.7	Undifferentiated	Mycalostyles 310–680 x 10–29	Anchorate unguiferous anisochelae 20–25	40–55	Sigmas 95–145	Barbados Trench and Mid Atlantic Ridge, bathyal- abyssal
*Cladorhiza microchela* Lévi, 1964	Lévi, 1964:73, Pl. IV, Fig. F	Stipitate erect, long stem with widely spaced perpendicular pinnate paired filaments on either side of Stem	55 × 0.5- 0.8	Axis of stem and filaments cored by longitudinal bundles of styles	Styles 1, up to 3200 × 45–50 styles 2, 1100- 1400 × 12–15	Undifferentiated	Not recorded	Tridentate unquiferous anisochelae 13–14	Absent	Absent	South China Sea, abyssal
*Cladorhiza oxeata* Lundbeck, 1905	Lundbeck, 1905:97; Hestetun, Tompkins- Macdonald & Rapp, 2017:33–34, Figs 26–27	Arbuscular erect, branched tree- like, solid branching main stem with side branches, both covered with numerous filaments, short basal Attachment	300 × 5–10	Axis of stem and branches composed of tightly connected bundles of oxeas, producing a smooth rigid central skeleton	Oxeas 390- (615)-845 x 4.9-(22.0)- 38.9	Undifferentiated	Undifferentiated	Multidentate anchorate anisochelae, 5 teeth, 22.6-(33.3)-42.0	26.7-(43.4)- 55.0	Sigmas 100-(125)- 153	Amphi-Atlantic, boreo-Arctic, mesophotic-bathyal
*Cladorhiza scanlonae* Goodwin, Berman, Downey & Hendry, 2017	Goodwin, Berman, Downey & Hendry, 2017:50–51, Fig. 8	Branching erect, short stem with rhizoid rootlet holdfast, body (upper stem) with short knobbly spicule column projections, terminations of branches with more pronounced nodules	80 × 3	Axis of main stem formed of columns of mycalostyles with bundles of ~ 15 spicules, one style in length, projecting at right angles, rootlets with columns of similar mycalostyles	Mycalostyles (upper branches) 1140–1495 x 24–47	Undifferentiated	Mycalostyles (rootlets) 857–1456 × 26–50	Unguiferate anisochelae 40–45	Absent	Absent	Sars Seamount, Drake Passage, Antarctica, mesophotic
*Cladorhiza tenuisigma* Lundbeck, 1905	Lundbeck, 1905:84, Pl. III, Fig. 2- 3, Pl. XII, Fig. 4; Koltun 1959:82, Fig. 37; Hestetun, Tompkins- Macdonald & Rapp, 2017:34–37 Fig. 28	Arbuscular erect, branching stem with side branches swollen at tips, both covered in long filaments projecting in all directions, basal stem anchored by rhizoid rootlets	150 x 2–5	Axis of stem and branches with bundles of mycalostyles forming a solid core, filaments cored by overlapping mycalostyles parallel to axial stem	Mycalostyles 430-(704)-918	Undifferentiated	Undifferentiated	Anchorate anisochelae, 5 teeth, 16.0-(23.4)-32.0	41.8-(49.5)- 58.1	Sigmas 31.4-(41.7)- 53.6	Iceland-Faroe Ridge up to Svalbard, mesophotic-bathyal
*Cladorhiza thomsoni* Topsent, 1909	Topsent, 1909:15, Pl. I, Fig. 7, Pl. II, Fig, 3; Hestetun, Fourt, Vacelet, Boury- Esnault & Rapp, 2015: 1337, Fig. 16	Arbuscular, branched tree- like, main axis carrying secondary filaments, filaments short, basal attachment unknown	20–90 × 0.5-7.5	Axis of stem composed of longitudinal bundles of mycalostyles in several concentric bands placed parallel to each other along the main axis, filaments with mycalostyles projecting tangentially from stem axis	Mycalostyles 1, 940–1230 x15.7–29.8 mycalostyles 2, 580–880 x 12.6–28.3	Undifferentiated	Not present	Anchorate anisochelae 27–34	Absent	Sigmas 1, 137–174, sigmas 2, (filaments) 41–61	Kane Fracture Zone, Mid-Atlantic Ridge & off Cape Town, SE Atlantic, bathyal-abyssal

### ***Cladorhiza vanessaekins* sp. nov.**

**Fig. 13:**
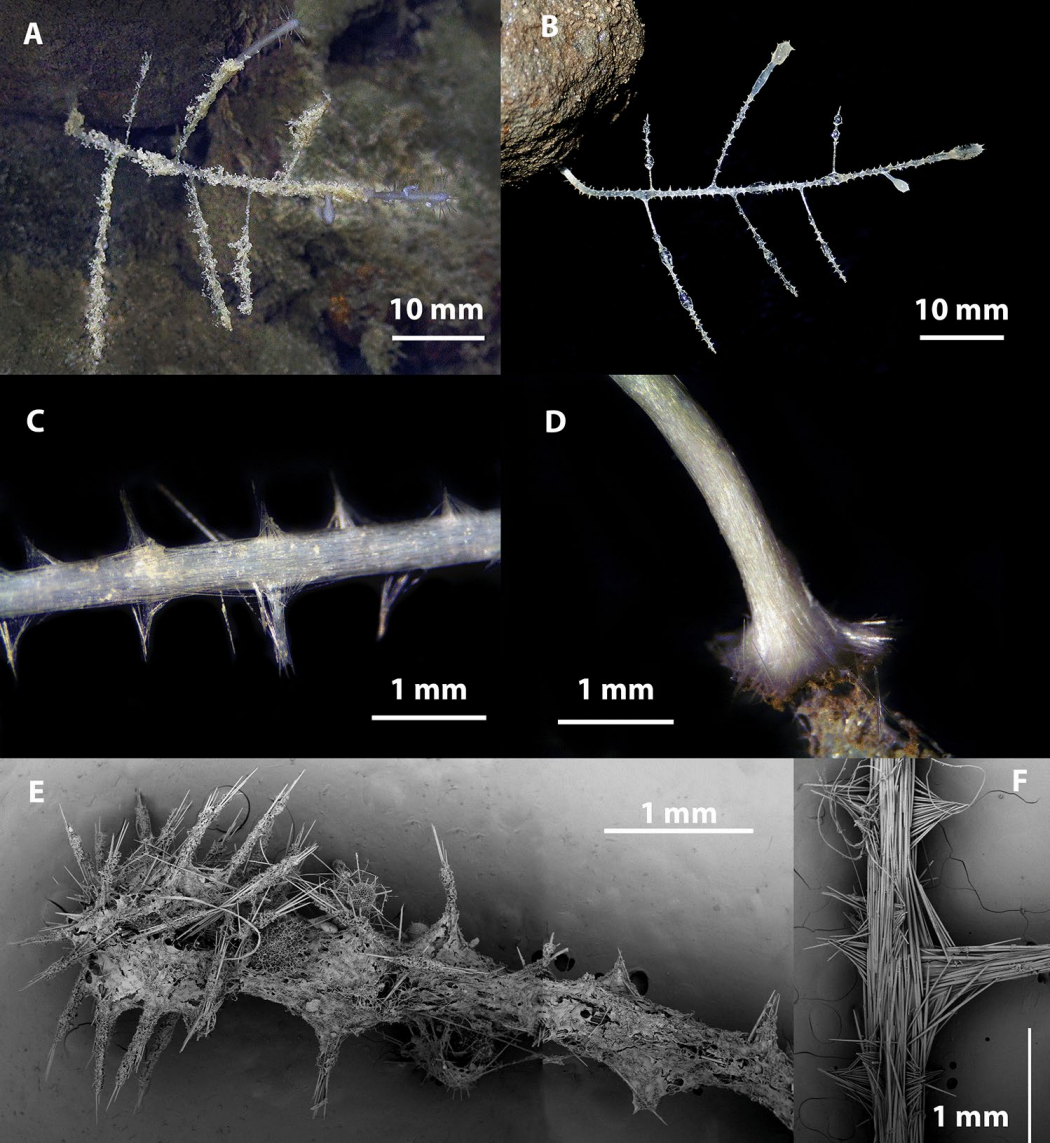
*Cladorhiza vanessaekins* sp. nov. (**A**). Holotype WAMZ100704 in situ before collection. (**B**). Holotype WAMZ100704 before preservation. (**C**). Stem showing the axial bundles of styles, with the filaments. (**D**). Basal holdfast showing the longitudinal mycalostyles. (**E**). SEM of one of the branches showing terminal swelling and filaments. (**F**). SEM showing attachment of the filaments and a branch to the stem axis.

**Fig. 14:**
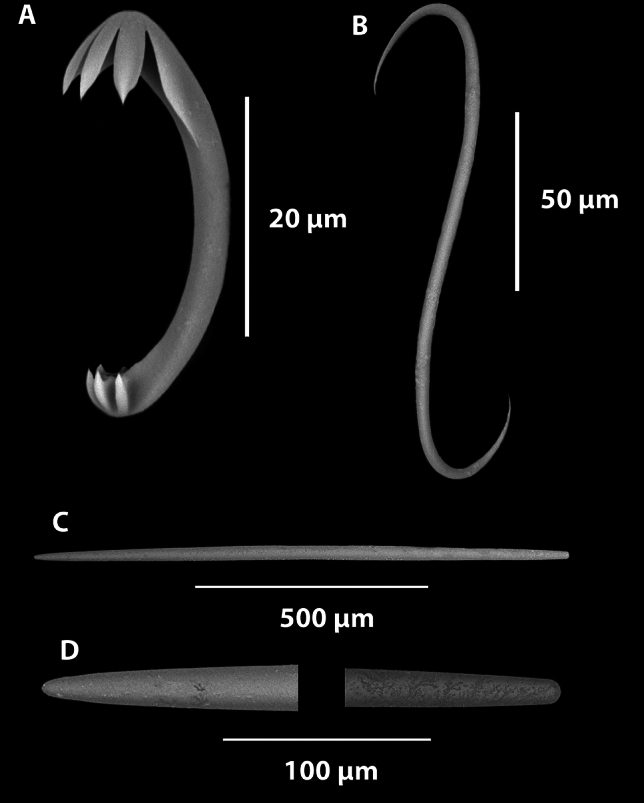
*Cladorhiza vanessaekins* sp. nov. (**A**). Anisochelae. (**B**). Sigma. (**C**). Mycalostyle. (**D**). Magnified ends of the mycalostyle illustrated in (**C**).

Figs. 6, 13, 14, Table 3.

urn:lsid:zoobank.org:act:21259137-0C02-4855-AE6D-EAB50CFE59A1

**Material examined**. Holotype: WAMZ100704, Cape Range Canyon, site CR10, Ningaloo region, Western Australia, Australia, 21°52′ 19″ S, 112°42′ 46″ E, 4435.9 m, dive S0343/016, Coll. Wilson, N., Rouse, G., Kirkendale, L. & Ritchie, J. on RV *Falkor* FK200308, 22 March 2020. Paratype: WAMZ100735, Cape Range Canyon, site CR13, Ningaloo region, Western Australia, Australia, 21°49′ 4”S, 112°30′ 39″ E, 4514.2 m, Dive S0347/013, Coll. Wilson, N., Rouse, G., Kirkendale, L. & Ritchie, J. on RV *Falkor* FK200308, 27 March 2020. 28S GenBank Accession [OR198144].

**Etymology**. Named after the first author’s sister Vanessa Ekins, in recognition of her invaluable service to the environment and her endeavours in revegetating riparian areas with native trees, to which this species represents arborescent morphology.

**Distribution**. This species is presently known only from the type and paratype localities in Cape Range Canyon, Ningaloo region, mid Western Australia, from abyssal depths.

**Description**. *Growth form*: An erect delicate single-axis arborescent sponge resembling a tree with seven alternate branches attached to a rock (Fig. 13A,B). The cylindrical stem is composed of four parallel columns of filaments. The sponge has a total length of 67 mm in height, the branches are up to 26 mm in length often with swelling at the terminal apices caused by multiple radiating filaments. The basal stem holdfast is 4 mm in diameter, while the stem is 0.6 mm in width (Fig. 13D). The branches are between 0.4 and 0.6 mm in width and up to 1.2 mm at the terminal swellings. Filaments occur between every 0.6 and 1 mm along both the stem and the branches. Each branch or stem may contain four alternate rows of filaments (Fig. 13C). The filaments are up to 1 mm long and 80 to 100 µm wide, however where the filament spicules diverge to form a supporting buttress against the stem, the buttress may be up to 600 µm wide.

*Colour*: Translucent white underwater, on-deck and when preserved in ethanol.

*Skeleton*: The outer layer is membranous and contains the abundant anisochelae and common large sigmas (Fig. 13E). It is ubiquitous over the whole sponge with the exclusion of the basal holdfast (Fig. 13D). This axis consists of bundles of mycalostyles longitudinally arranged (Fig. 13C,F). The branches and filaments also consist of longitudinally arranged mycalostyles, with radial buttressed arrangement for support against the stem. The basal holdfast also consists of the same mycalostyles bundled together as in the stem (Fig. 13D).

*Megascleres*: The large mycalostyles (556-(864)-2510 x 7.4-(21.4)-44.9 µm, n=76) are fusiform, with an abruptly sharp tip with the greatest width at the centre of the spicule (Fig. 14C,D). The same continuous mycalostyles occur in the stem, filaments and basal root attachment of the sponge. Similarly for the paratype the sizes are (528-(704)-1050 x 7.8-(17.8)-32.7 µm, n=55).

*Microscleres*: These consist of a single type and size class of unguiferate anisochelae and large sigmas. The anisochelae (size range 22.0-(27.4)-33.7 x 1.8-(2.5)-3.5 µm, n=37) have five upper alae, and five lower alae (Fig. 14A). Similarly in the paratype WAMZ100735 the anisochelae sizes were (26.0-(28.9)-32.0 x 1.3-(2.6)-4.2 µm, n=48). The large sigmas are very thin and sharp with contortion of approximately 90-120° (Fig. 14B), the sizes are (66-(135)-168 x .21-(4.1)-5.7 µm, n =24) and for the paratype (112-(138)-161 x 2.1-(4.1)-6.3 µm, n=41).

*Molecular data*: Sequence data was produced from WAMZ100735, which was sister to a clade containing all the other included species of *Cladorhiza* (Fig 6).

**Remarks**. Despite the appearance of the holotype looking like *Cladorhiza abyssicola*, the new species has larger mycalostyles, and it also lacks both the smaller size class of sigmas and sigmancistras. It also differs from *Cladorhiza abyssicola* by being attached to a rock by a holdfast rather than rhizoids anchoring in the mud. The northern hemisphere species *Cladorhiza abyssicola* has been well reviewed^26^. The new species is similar to one from the south eastern Pacific i.e. *Cladorhiza segonzaci* Vacelet 2006. However, the much smaller anisochelae of *C. segonzaci* only have three lower alae, smaller sigmas and the presence of sigmancistras. *Cladorhiza microchela* Lévi, 1964, from the South China Sea (4330 m) has very small anisochelae, not exceeding 13 µm, it too has an absence of sigmas and sigmancistras. This new species morphologically resembles *Chondrocladia* (*Chondrocladia*) *zygainadentonis* (see also^24^). However, it lacks the isochelae which would place it in that genus, and the molecular data supports its placement in *Cladorhiza*.

### Genus *Nullarbora* Ekins, Erpenbeck, Goudie & Hooper, 2020

**Diagnosis**. Cladorhizidae with anchorate anisochelae and a pinnate body shape, i.e. filaments at right angle to a straight stem.

**Type species**. *Nullarbora heptaxia* Ekins, Erpenbeck, Goudie & Hooper, 2020b.

**Remarks**. There are currently 14 accepted species in this genus.

### ***Nullarbora ningalooa* sp. nov.**

Figs. 6, 15, 16.

**Fig. 15:**
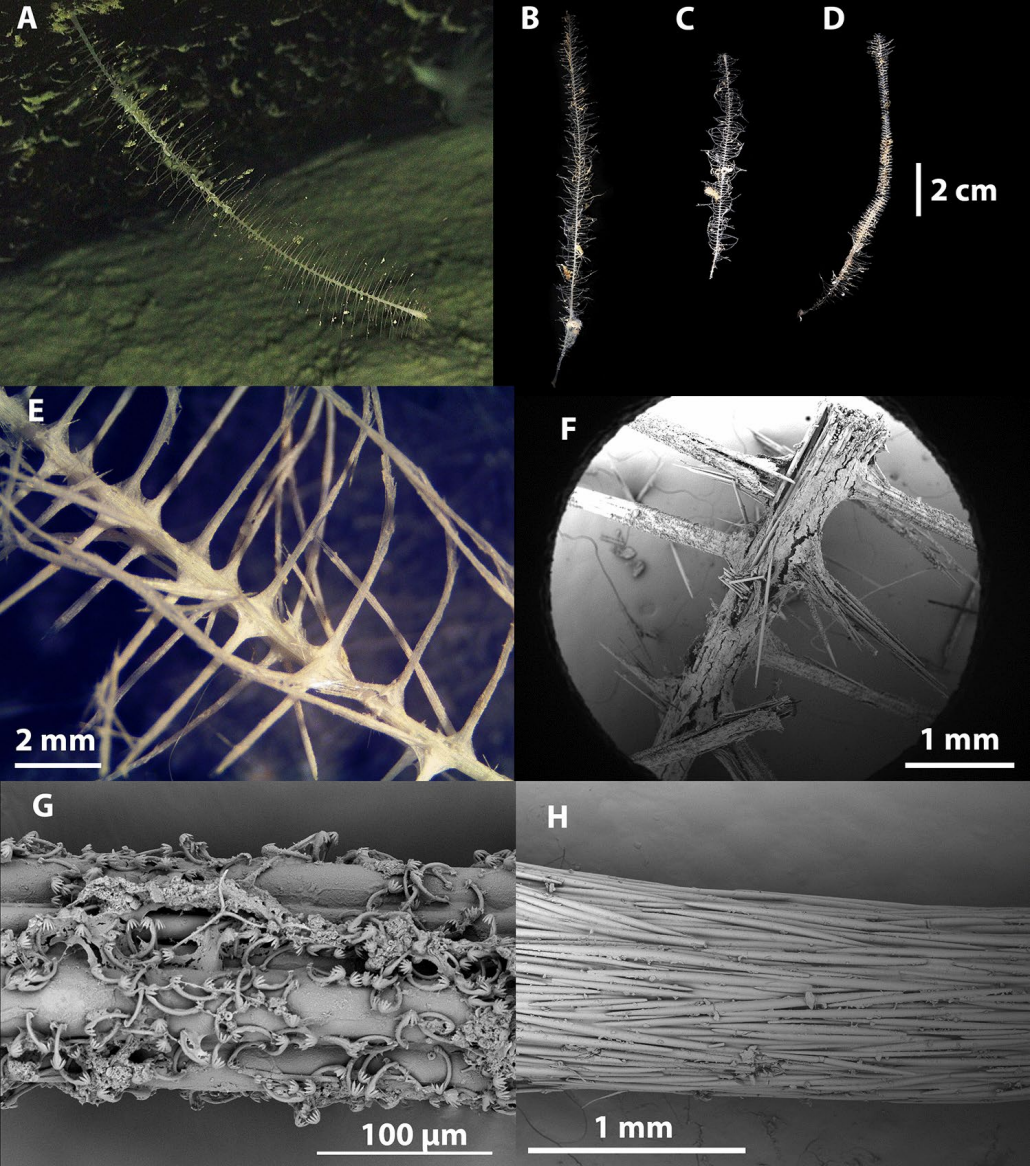
*Nullarbora ningalooa* sp. nov. (**A**). Holotype WAMZ100670 in situ before collection. (**B**). Holotype WAMZ100670 before preservation. (**C**). Paratype WAMZ100671 before preservation. (**D**). Paratype WAMZ100685 before preservation. (**E**). Stem showing axial bundles of styles. (**F**). SEM showing attachment of the filaments to the stem axis. (**G**). Filaments showing the encrusting anisochelae. (**H**). Lower stem showing the thick longitudinal mycalostyles.

**Fig. 16:**
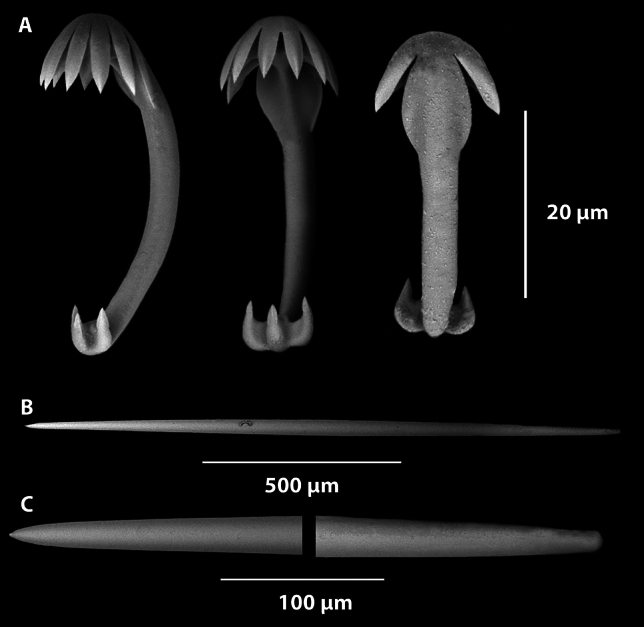
*Nullarbora ningalooa* sp. nov. (**A**). Unguiferate anisochelae. (**B**). Mycalostyles. (**C**). Magnified ends of the mycalostyle illustrated in (**B**).

urn:lsid:zoobank.org:act:D5504B82-999A-45F7-89D7-C5BDF741A7F8

**Material examined**. Holotype: WAMZ100670, Cape Range Canyon site CR7, Ningaloo region, Western Australia, Australia, 21°53′ 52″ S, 112°54′ 19″ E, 2914.22 m, Dive S0338/012, Coll. Wilson, N., Rouse, G., Kirkendale, L. & Ritchie, J. on RV *Falkor* FK200308, 17 March 2020. 28S GenBank Accession [OR198145]. Paratypes: WAMZ100671, same collection details as WAMZ100670. 28S GenBank Accession [OR198146]. WAMZ100685, Cape Range Canyon, site CR9, Ningaloo region, Western Australia, Australia, 21°51′ 27″ S, 112°4525″ E, 3732.96 m, Dive S0340/022, Coll. Wilson, N., Rouse, G., Kirkendale, L. & Ritchie, J. on RV *Falkor* FK200308, 19 March 2020. 28S GenBank Accession [OR198147].

**Etymology**. Named after Ningaloo, the region in mid Western Australia from which the species was collected.

**Distribution**. This species is presently known only from the type and paratype localities in the Cape Range Canyon, Ningaloo region, mid Western Australia, from abyssal depths.

**Description**. *Growth form*: Stipitate, erect, unbranched, tall, bottle-brush shaped sponge adorned with copious fragile horizontal filaments (Fig. 15A). The holotype is 180 mm in length and the stem is 17 mm in length and 1 mm in width. There are usually four (sometimes five) columns of 98 rows of filaments. The filaments are up to 10 mm in length and 0.1-0.25 mm in width. This species also has basal holdfast 6 mm in diameter. Both paratypes are smaller and thinner of 105 and 135 mm in length and the filaments only reach a maximum of 13-15 mm.

*Colour*: Off-white underwater, on deck and when preserved in ethanol.

*Skeleton*: This outer layer is membranous and contains the abundant anisochelae. This is clearly seen on the filaments (Fig. 15G). The anisochelae are absent in the outer layert of the lower stem and root holdfast (Fig. 15H). In cross-section the stem has a radial skeleton, with a very small core. Around the nucleus four to five arms radiate outwards (Fig. 15E,F). These filaments are composed of thin bundles of longitudinal mycalostyles, providing support to the sponge by diverging where they meet the stem.

*Megascleres*: The large mycalostyles (136-(1180)-1760 x 5.5-(32.3)-71.5 µm, n=97) are fusiform, with an abruptly sharp tip with the greatest width at the centre of the spicule (Fig. 16B,C, Table 1). The same continuous styles occur in the stem, filaments and basal root attachment of the sponge. Similarly for the paratypes the sizes are (454-(993)-1440 x 8.3-(25.8)-49.9 µm, n=42) for WAMZ100671 and (516-(977)-1790 x 12.3-(26.3)-45.0 µm, n=28) for WAMZ100685.

*Microscleres*: These consist of a single type and size class of unguiferate anisochelae (Fig 16A). The anisochelae (size range 25.0-(30.7)-40.0 x 1.3-(2.6)-3.9 µm, n=34) have seven upper alae, and three teeth on the lower alae. Similarly in the paratype WAMZ100671 the anisochelae is 26.3-(29.8)-32.6 x 1.3-(2.2)-3.1 µm, n=41) and in paratype WAMZ100685 the sizes are (26.7-(29.8)-33.6 x 1.7-(2.8)-3.7 µm, n=38).

*Molecular data*: The three specimens sequenced here share the same 28S C-region haplotype and show a sister relationship to the other included species of *Nullarbora* (Fig 6).

**Remarks**. This species is unique amongst the Cladorhizidae having stipitate bottle-brush growth form, a single type and size class of anisochelae and an absence of sigma and sigmancistras. These anisochelae and the absence of sigmas and sigmancistras differentiate *N*. *ningalooa* sp. nov. from four morphologically similar species. *Nullarbora rectangularis* (Ridley & Dendy, 1887) and *N. evae* (Lundsten, Reiswig & Austin, 2014) both have a single size category of multidentate unguiferate anchorate anisochelae, while *N. caillieti* (Lundsten, Reiswig & Austin, 2014) and *N. investigator* (Ekins, Erpenbeck & Hooper, 2020a) have two size categories of anisochelae but have unguiferate anchorate (see Table 5^5^). This new species is most similar morphologically to *Nullarbora heptaxia* but it lacks the larger second anisochelae, and it only has four to five radials on the axis unlike seven in *N. heptaxia*. Species within Cladorhizidae with the stipitate bottle brush like morphology i.e. *Nullarbora* species are tabulated in Table 5^5^. This new species differs from *N. evae*, by having 7 teeth on the head of the chelae instead of five. The differences between *N. evae* and *N. rectangularis* (especially when Koltun’s measurements are included^25,27^), reduce the difference to the presence of sigmancistras and a second size groups of sigmas.

## Discussion

The addition of these six new species in the present work brings the known fauna of Cladorhizidae in Australian waters to 41. All the carnivorous sponges in Australian waters were previously compared in Ekins et. al. (^28^, Table 1). That included the recent description of 17 new species from off the Queensland and New South Wales coastlines^4^, three new species from the Great Australian Bight^5^, a new species from Northern Queensland^28^ and three new species from the Great Barrier Reef^24^.

The long-standing Cladorhizidae genera have been *Abyssocladia* Lévi, 1964, *Asbestopluma* Topsent, 1901, *Axoniderma* Ridley & Dendy, 1886, *Chondrocladia* Thomson, 1873, *Cladorhiza* Sars, 1872, *Euchelipluma* Topsent, 1909 and *Lycopodina* Lundbeck, 1905, some of which were all illustrated in Figure 2 of ^21^. With the more recent addition of new genera *Abyssosdiskos* Ekins et al., 2020b, *Australocladia* Kelly & Vacelet, 2023, *Bathytentacular* Ekins et al., 2020b, *Cercicladia* Ríos et al., 2011, *Koltunicladia* Hestutun et al., 2016a, *Lollipocladia* Vacelet, 2008, and *Nullarbora* Ekins et al., 2020b and *Patriciacladia enigmatica* Kelly et al. 2023, it is clear that further diversity is still forthcoming.

The genus* Abyssocladia* currently contains 38 accepted species^22^. Within Australia there are seven *Abyssocladia* species. *Abyssocladia desmophora* was the first species discovered in Australia. This was recently followed by three new species described from off the Queensland and New South Wales coastlines, *A. annae*, *A. escheri* and *A. gliscofila*, and one from the Great Australian Bight *Abyssocladia oxyasters,* as well as two new ones from the Great Barrier Reef *Abyssocladia falkor* and *Abyssocladia jeanvaceleti*.

The addition of molecular data from *A. escheri* and *A. gliscofila* (in Figure 3 of ^4^) showed them to be outliers from the remaining sequenced *Abyssocladia* species. Despite the fact that these were pinnate species, with the small number of species available it was not prudent to separate these into a new genus. The addition of *A. oxyasters* (Figure 1 of ^5^), also re-emphasized the maintenance of *Abyssocladia* as a single genus. Unfortunately, as with much sponge taxonomy, the type species of the genus, *A*.* bruuni*, from the Kermadec Trench, has not yet been sequenced, nor has a recent sample of it been recovered to help diagnose the *Abyssocladia* genus.

The new species *Axoniderma challengeri* sp. nov. joins the recently resurrected genus^5^. This raises the number of species within this genus to 11, with species in Australia also including *A. australis* (Ekins, Erpenbeck & Hooper, 2020), *A. poritea* and *A. wanda*.

The two new specimens of *Cladorhiza vanessaekins* sp. nov. described here belong to the arboresent group, which include the closely related *C. abyssicola*, the type species, with which it bears morphological similarity. They differ in the spicule composition and the size of the spicules, establishing them as a new species. *Cladorhiza vanessaekins* sp. nov also differs by having a holdfast and is attached to a rock, as opposed to *C. abyssicola* which has branched rhizome roots, with the exception of a single specimen from Tenerrife (Topsent, 1909). The new species occurs in the southern Indian Ocean is also very geographically distant from the North Atlantic and the Mediterranean Sea, where *C. abyssicola* appears to be fairly common.

*Nullarbora*, a genus recently split off from *Cladorhiza*^5^ contained two Australian species *N. investigator* and *N. heptaxia*. The addition of this new species *Nullarbora ningalooa* sp. nov. brings the total number of species of this pinnate genus to 13 (three in Australia).

The continuing discovery of new species of Cladorhizidae is a direct result of the increasing amount of exploration undertaken at the bathyal and abyssal depths. This activity is currently being advanced by the availability of ROV’s and other deep-sea research vessels. Similarly, the taxonomy of the group has been assisted and expedited by the application of molecular techniques. The establishment of new species and new genera, however, is still hampered by the usual problems with modern sponge taxonomy i.e. the lack of DNA obtainable from type material, especially applicable to these tiny species located several thousand metres below sea level.

## Additional references in authorities and tables

- Carter, H. J. Descriptions and Figures of Deep-Sea Sponges and their Spicules, from the Atlantic Ocean, dredged up on board H.M.S.‘Porcupine’, chiefly in 1869 (concluded). *Ann. Mag. Nat. Hist.,* (4) **18**(105): 226*–*240; (106): 307*–*324; (107): 388*–*410;(108): 458*–*479, pls XII*–*XVI (1876).
- Dendy A. On a remarkable new species of Cladorhiza obtained by H.M.S. ‘Challenger’. Ann. Mag. Nat. Hist., 20, 279–282 (1887).
- Dendy, A. Report on the Sigmatotetraxonida collected by H.M.S. ‘Sealark’ in the Indian Ocean. In: Reports of the Percy Sladen Trust Expedition to the Indian Ocean in 1905, Vol. 7. Trans. Linn. Soc. London, 18(1), 1–164, pls 1–18 (1922). https://doi.org/10.1111/j.1096-3642.1922.tb00547.x
- Göcke, C. Hestetun, J. T. Uhlir, C. Freiwald, A. Beuck, L. Janussen, D. Cladorhiza corallophila sp. nov., a new carnivorous sponge (Cladorhizidae, Demospongiae) living in close association with Lophelia pertusa and Madrepora oculate (Scleractinia). Zootaxa, 4168(3), 512–524 (2016). https://doi.org/10.11646/zootaxa.4168.3.4
- Goodwin, C. E. Berman, J. Downey, R. V. Hendry, K. R. Carnivorous sponges (Porifera: Demospongiae: Poecilosclerida: Cladorhizidae) from the Drake Passage (Southern Ocean) with a description of eight new species and a review of the family Cladorhizidae in the Southern Ocean. Invert. Syst., 31(1), 37–64 (2017). https://doi.org/10.1071/IS16020
- Gray, J. E. Notes on the Arrangement of Sponges, with the Descriptions of some New Genera. Proc Zool. Soc. Lond. 1867(2), 492–558, pls XXVII–XXVIII (1867).
- Hentschel, E. Monaxone Kieselschwämme und Hornschwämme der Deutschen Südpolar-Expedition 1901-1903. Deutsche Südpolar-Expedition. 15(1), 35–141, pls IV–VIII (1914).
- Hestetun, J., Fourt, M.; Vacelet, J.; Boury-Esnault, N.; Rapp, H. T. Cladorhizidae (Porifera, Demospongiae, Poecilosclerida) of the deep Atlantic collected during Ifremer cruises, with a biogeographic overview of the Atlantic species. J. Mar. Biol. Assoc. U. K., 95(7), 1311–1343 (2015). https://doi.org/10.1017/S0025315413001100
- Hestetun, J. T., Rapp, H. T. & Pomponi, S. Deep-Sea Carnivorous Sponges from the Mariana Islands. Front. Mar. Sci., 6, 371 (2019). https://doi.org/10.3389/fmars.2019.00371
- Hooper, J. N. A. & Lévi, C. Esperiopsis desmophora n.sp. (Porifera: Demospongiae): a desma-bearing Poecilosclerida. Mem. Queensl. Mus., 27(2), 437–441 (1989).
- Ise, Y., & Vacelet, J. New carnivorous sponges of the genus Abyssocladia (Demospongiae, Poecilosclerida, Cladorhizidae) from Myojin Knoll, Izu-Ogasawara Arc, southern Japan. Zool. Sci. 27(11), 888–894 (2010).
- Kelly, M., Vacelet, J., Hestetun, J., & Mills, S. New species of Abyssocladia and two new cladorhizid genera (Porifera, Cladorhizidae) from New Zealand and Australia. Zootaxa 5270(1), 1–47 (2023). https://www.mapress.com/zt/article/view/zootaxa.5270.1.1
- Koltun, V. M. Sponge fauna of the northwestern Pacific from the shallows to the hadal depths. Pp. 165–221. In Fauna of the Kurile-Kamchatka Trench and its environment. Institute of Oceanology of the Academy of Sciences of the U.S.S.R., 86. (Akademiya Nauk SSSR. Trudy Instituta Okeanologii in P.P. Shishov and Izdatelstvo Nauka, Moskwa) (ed. Bogorov, V.G.) 1–372, pls 1–8 (1970).
- Laubenfels, M. W. de. A Discussion of the Sponge Fauna of the Dry Tortugas in Particular and the West Indies in General, with Material for a Revision of the Families and Orders of the Porifera. Publ. - Carnegie Instit. Wash. 467 (Tortugas Laboratory Paper 30) 1–225, Pls. 1–22 (1936).
- Lehnert, H., Watling, L. & Stone, R. Cladorhiza corona sp. nov. (Porifera: Demospongiae: Cladorhizidae) from the Aleutian Islands (Alaska). J. Mar. Biol. Assoc. U. K., 85, 1359-1366 (2005). https://doi.org/10.1017/S0025315405012531
- Lundsten, L., Reiswig, H. M. & Austin, W. C. Four new species of Cladorhizidae (Porifera, Demospongiae, Poecilosclerida) from the Northeast Pacific. Zootaxa, 3786, 101–123 (2014). https://doi.org/10.11646/zootaxa.3786.2.1
- Ríos, P., Kelly, M., Vacelet, J. Cercicladia australis, a new carnivorous sponge with novel chelae from the Tasman Basin and the Argentine Patagonian Margin (Porifera, Cladorhizidae). Zootaxa 3131, 52–62 (2011). https://doi.org/10.11646/zootaxa.3131.1.3
- Sars, G. O. On some remarkable forms of animal life from the great deeps off the Norwegian coast. Part 1, partly from posthumous manuscripts of the late prof. Mich. Sars. University Program for the 1rs half-year 1869. Brøgger & Christie, Christiania viii + 82 pp., pls 1–6. (1872). available online at http://biodiversitylibrary.org/page/11677777
- Thomson, C. W. The Depths of the Sea. 527 pp., (Macmillan and Co., London, 1873). available online at https://www.biodiversitylibrary.org/page/1633031
- Topsent, E. Spongiaires. Résultats du voyage du S.Y. ‘Belgica’ en 1897–99 sous le commandement de A. de Gerlache de Gomery. Expédition antarctique belge. Zoologie, 4, 1–54, pls I–VI (1901). https://doi.org/10.5962/bhl.part.18721
- Vacelet, J. New carnivorous sponges (Porifera, Poecilosclerida) collected from manned submersibles in the deep Pacific. Zool. J. Linn. Soc, 148, 553–584 (2006). https://doi.org/10.1111/j.1096-3642.2006.00234.x
- Vacelet, J. A new genus of carnivorous sponges (Porifera: Poecilosclerida, Cladorhizidae) from the deep N-E Pacific, and remarks on the genus Neocladia. Zootaxa 1752, 57–65 (2008). https://doi.org/10.11646/zootaxa.1752.1.3
- Vacelet, J. & Boury-Esnault, N. A new species of carnivorous deep-sea sponge (Demospongiae: Cladorhizidae) associated with methanotrophic bacteria. Cah. Biol. Mar. 43, 141–148 (2002).

## Data Availability

All data generated or analysed during this study are included in this published article (and its Supplementary Information files), and from GenBank under accession numbers OR198144- OR198148.
